# Revisiting molecular descriptors with TDiMS for interpretable intramolecular interactions based on substructure pairs

**DOI:** 10.1038/s43588-026-01036-3

**Published:** 2026-09-17

**Authors:** Lisa Hamada, Akihiro Kishimoto, Kohei Miyaguchi, Masataka Hirose, Junta Fuchiwaki, Indra Priyadarsini, Seiji Takeda

**Affiliations:** 1 IBM Research, Chuo-ku, Japan; 2 JSR Corporation, Minato-ku, Japan

**Keywords:** Cheminformatics, Computational methods

## Abstract

Molecular descriptors play a crucial role in representing the structural features of molecules for machine learning-based physical property prediction. However, current descriptors either consider only local aspects of molecular structures or fail to effectively learn nonlocal structural features involving long-distance intramolecular interactions. Here, to address this issue, we present a descriptor named TDiMS. TDiMS effectively summarizes the enumerated pairwise topological distances between molecular substructures, thus capturing nonlocal interactions. Our evaluation shows that TDiMS successfully identifies essential features of large structures and outperforms other representative descriptors in predicting properties for which distances between substructures are a primary factor. In addition, these identified features are highly interpretable for experts in materials discovery.

## Abstract (web summary)

A descriptor, TDiMS, is developed to summarize the enumerated pairwise topological distances between molecular substructures and identify interpretable molecular features for materials discovery.

## Main

Materials informatics has advanced materials science over the past two decades^1^, focusing on reducing time and cost and increasing the variability of materials discovery. Predicting chemical or physical properties is a core task in materials design and is often addressed through quantitative structure–property relationship modeling^2^.

Descriptors are key components of the quantitative structure–property relationship that consist of numerical chemical features systematically extracted from molecules in simplified molecular input line entry system (SMILES)^3^ representation. These features, which are represented as a feature vector, are passed to a machine learning (ML) estimator that outputs a predicted value for a target property. During the training phase, the model parameters of the ML estimator are updated. Once training is complete, the model is applied to predict the property values of the target molecules.

Feature representation depends on the descriptor and must meet two requirements^4^. First, the features must reflect essential molecular characteristics for developing an effective property prediction model. This is especially difficult for large molecules because of their complex intramolecular interactions on nonlocal substructures. Second, the features must be interpretable, enabling experts to understand the results predicted by the model. The development of an effective descriptor that meets both requirements remains a challenge.

### Contributions

On the basis of molecular substructure pairs, we introduce a general-purpose descriptor. From both the ML and materials science perspectives, we perform analyses with four datasets of practical size, including the Chromophore datasets^5^, which contain large, complex molecular structures. Our contributions are summarized as follows:

1. Topological distance of intramolecular substructures (TDiMS) is designed to overcome the limitations of existing descriptors that either capture only the local features of large molecules or compromise interpretability.
2. Performance comparisons of property predictions with various representative descriptors clearly reveal that TDiMS outperforms other descriptors for large molecules.
3. Our analysis revealed the existence of a quantitative threshold regarding whether TDiMS performed effectively or not.
4. There is strong evidence that TDiMS successfully extracts important nonlocal features that other methods fail to capture.
5. TDiMS features are regarded as important concepts in materials science, particularly those related to physical energies and the spatial alignment of chromophores. These concepts can be applied in the design of energy devices such as biosensors, organic electroluminescence devices and solar cells.

### Background on SMILES-based molecular descriptors

There are several approaches to creating SMILES-based descriptors. For example, knowledge-driven descriptors are designed to reflect basic properties and knowledge, such as XLogP^6^. These descriptors focus only on properties that are very similar to these basic properties. Enumerative descriptors exhaustively enumerate substructure patterns that appear in a molecule and use them as general-purpose features. For example, fingerprint descriptors count predefined local substructures^7,8^, whereas other descriptors enumerate all possibly distant pairs of predefined substructures to capture nonlocal information^9,10^. In general, enumerative descriptors substantially increase the number of enumerated patterns as the dataset size increases. Although considering only small patterns^9^ or leveraging hashing^7,10^ can mitigate this issue, accuracy and interpretability are compromised. Neural network (NN)-based descriptors rely on NNs that automatically exploit feature representations^11,12^. Without the need for molecule-specific knowledge or labels, these NNs are pretrained in a self-supervised manner with million- or billion-scale public datasets. Despite recent promising results, their feature representations do not provide any physical insights because of their black-box nature.

An additional challenge for creating effective descriptors is that the target property datasets used to train ML estimators are limited to specific molecules. For example, one proprietary dataset includes only 91 sugar molecules and 250 dye molecules^13^.

TDiMS is based on topological distances (TDs) between enumerated substructure pairs. Unlike other enumerative descriptors, TDiMS removes redundant pairs, allowing it to handle larger substructure pairs located far from each other without any information loss. Thus, while ensuring interpretability, TDiMS achieves better accuracy than existing descriptors do, especially with a training dataset of practical size with large molecules, as indicated by our performance evaluation. Our ML-based feature contribution analysis and materials science interpretations of substructure pairs also clearly demonstrate these advantages of TDiMS.

## Results and discussion

### TDiMS framework

**Fig. 1:**
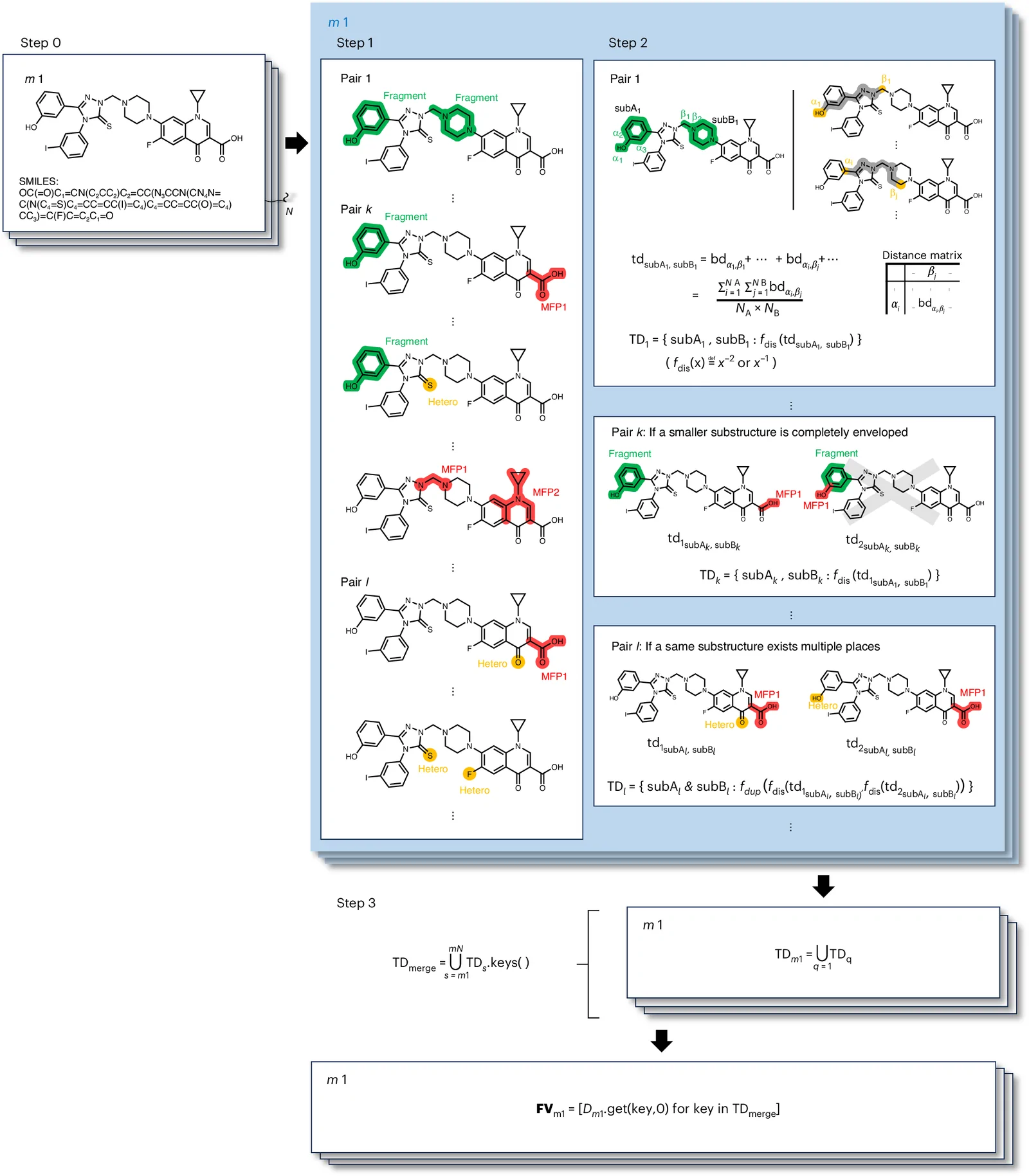
TDiMS workflow. The workflow consists of receiving a list of molecules in SMILES (step 0), extracting a feature set with duplicate detection of substructure pairs (steps 1 and 2) and embedding the molecules into their feature vectors on the basis of the features of all the molecules in the dataset (step 3). MFP1 and MFP2 denote circular substructures derived from the Morgan fingerprint with radii of 1 and 2, respectively.

Figure 1 shows the algorithm workflow. The process begins by receiving a dataset of molecules represented in SMILES format (step 0). Given a set of substructures to consider, TDiMS enumerates all substructure pairs in each molecule (step 1) and calculates the TD for each pair (pair 1, step 2). This enumeration step yields a potentially large number of pairs, which may become impractical. To address this problem, TDiMS removes pairs on the basis of structural inclusion relations (pair *k*, step 2) and aggregates the distances of identical pairs in different locations (pair *l*, step 2). After processing the dataset, TDiMS generates feature vectors for target molecules (step 3). The feature vector size depends on the dataset but is standardized across molecules. See the ‘TDiMS details’ section in the Methods for the complete procedures.

### Predictive performance and analysis of TDiMS

#### Comparing predictive performance as a standalone descriptor

After creating three datasets with various properties and molecular structure sizes (see ‘QM9 and Chromophore datasets’ section in the Methods), we evaluated the *R*^2^ scores of the prediction models that receive input feature vectors derived from the descriptors and that return prediction values. The five representative descriptors were selected as baselines: one knowledge-driven descriptor for comprehensive physical chemistry (Mordred^14^), two high-performing NN-based descriptors (MoLFormer^11^ and MolCLR^12^) based on generative chemical language models (CLMs) and graph NNs (GNNs), and two enumerative descriptors based on intramolecular TDs (Atom-Pair^9^ and MAP4^10^). See the ‘Baseline descriptors’ and ‘Prediction model’ sections in the Methods for details.

Table 1 shows the *R*^2^ scores of the best-performing models. Each descriptor was used as a standalone descriptor for property prediction. See Supplementary Fig. 3 for their corresponding parity plots.

**Table 1:** Performance of each descriptor used as a standalone to perform six prediction tasks on datasets of 1,000 molecules

Dataset	Crm. (CHCl_3_)		Crm. (CH_3_OH)		QM9	
Structure size	Large		Large–medium		Small	
Property	${\mathbf{\lambda }}_{{\mathbf{abs}}.}^{\mathbf{\max}}$	Δ*λ*	${\mathbf{\lambda }}_{{\mathbf{abs}}.}^{\mathbf{\max}}$	Δ*λ*	HOMO	LUMO
MoLFormer	0.792 ± 0.218^*^	0.500 ± 0.185^*^	0.880 ± 0.066^*^	0.402 ± 0.054	0.612 ± 0.044^*^	**0.906** ± 0.013^*^
MolCLR	0.739 ± 0.046^*^	0.482 ± 0.065^*^	0.781 ± 0.045^*^	0.437 ± 0.065^*^	**0.651** ± 0.049^*^	0.862 ± 0.036^*^
Mordred	0.832 ± 0.021	0.490 ± 0.048	0.816 ± 0.036	0.456 ± 0.049	0.631 ± 0.309	0.852 ± 0.057
Atom-Pair	0.823 ± 0.027	0.557 ± 0.042	0.837 ± 0.029	0.503 ± 0.043	0.520 ± 0.041	0.774 ± 0.024
MAP4	0.725 ± 0.028	0.504 ± 0.036	0.787 ± 0.030	0.470 ± 0.040	0.161 ± 0.033	0.256 ± 0.026
**TDiMS**	**0.875** ± **0.019**	**0.600** ± **0.052**	**0.883** ± **0.015**	**0.574** ± **0.057**	0.547 ± 0.038	0.732 ± 0.029

Values are presented as mean *R*^2^ ± standard deviation (s.d.) across 30 test folds obtained from 3-fold cross-validation with 10 repeats. Bold indicates the top-performing model for each task. Notably, in predicting Δ*λ*, TDiMS achieved 7.7% and 14.1% improvements over the second-best scores for solvents CHCl_3_ and CH_3_OH, respectively. Crm. stands for Chromophore, with the type of solvent indicated in parentheses. For NN-based descriptors, the better score between embedding-only and fine-tuned settings is reported. An asterisk denotes results obtained with fine-tuned settings, which indicates that fine-tuned versions performed better in most of the cases. See Supplementary Table 4 for each setting of the NN-based descriptors.

TDiMS outperformed the other descriptors across all prediction tasks on the Chromophore dataset, although fine-tuned MoLFormer was competitive in predicting ${\lambda }_{{\rm{abs}}.}^{\max }$ with CH_3_OH. These results demonstrate the strength of TDiMS in handling larger molecular structures. See Supplementary Information section 2.2 for detailed discussions.

Supplementary Information section 2.3 shows that TDiMS was robust even if the dataset size was reduced to 200. Supplementary Information section 2.4 shows that fine-tuning of NN-based descriptors did not always lead to improvements.

#### Effectiveness of TDiMS when combined with other descriptors

**Extended Data Fig. 1:**
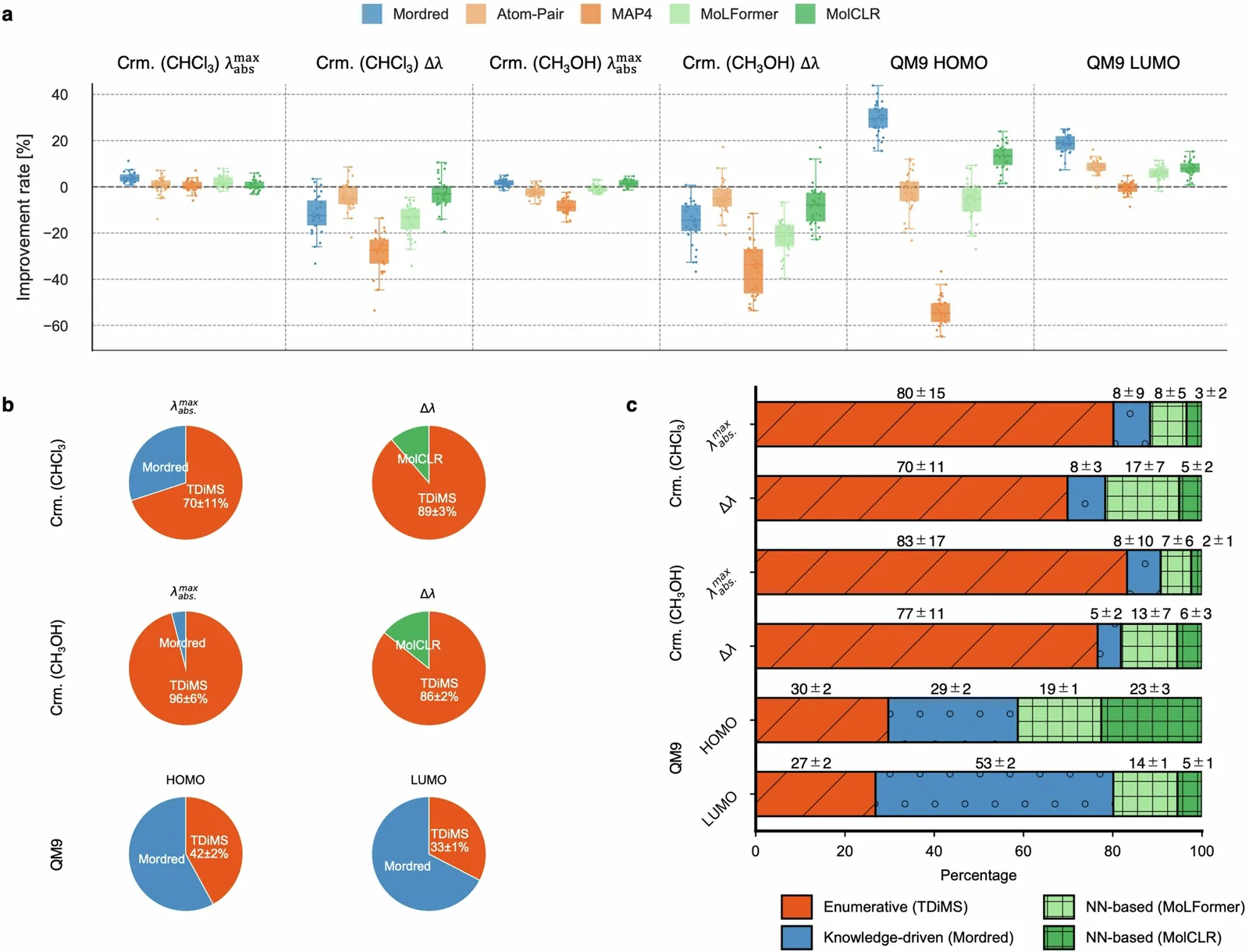
Analysis of TDiMS concatenated with other descriptors. **a**, Box-and-whisker plots of the performance improvement rate (%) of TDiMS plus each descriptor over TDiMS alone across 30 test folds obtained from 3-fold cross-validation with 10 repeats. Boxes, centre lines, whiskers, and points indicate interquartile ranges (IQRs), medians, 1.5 × IQR, and individual test folds, respectively. **b**, Proportion of the gSHAP value for TDiMS features compared to the descriptor that achieved the best score when concatenated with each best-performing LASSO model. **c**, Proportion of the gSHAP value for each descriptor type when all descriptors are concatenated. Feature importance was summed by each descriptor type: enumerative (TDiMS), knowledge-driven (Mordred), and NN-based (MoLFormer and MolCLR) descriptors. MoLFormer and MolCLR are the embedding-only versions. For **b** and **c**, we performed bootstrap sampling and created ten datasets of 1000 molecules from the original dataset for each prediction task, and the mean and SD were calculated across these ten trials. In this way, even though each dataset may contain duplicate molecules, bootstrap sampling ensures the stability of the trained parameters and the reliability of the resultant analysis. Source data.

As a well-known performance enhancement, we compared the predictive performance using concatenated feature vectors of TDiMS and another descriptor as input to an estimator. The improvement achieved by concatenation indicates that additional features of the concatenated descriptor provide beneficial information not captured by TDiMS alone. Extended Data Fig. 1a shows the relative improvement over standalone TDiMS in terms of the *R*^2^ scores. Concatenation improved performance by more than 18% for QM9 in the case of Mordred but by less than 4% for Chromophore across all descriptors, suggesting that TDiMS already captured most useful information in Chromophore. As discussed in the previous subsection, these findings are reasonable: TDiMS focuses on long-distance pairwise substructures and has difficulty providing useful features for such small molecules.

We further analyzed the importance of the descriptors via Shapley additive explanations (SHAP)^15^, a standard method used to explain the predictions, with bootstrap sampling^16^. In each trained prediction model, SHAP additively partitions the prediction value into SHAP values, which represent the contributions of individual features (see ‘Computation of feature importance’ section in the Methods). Specifically, we first computed the importance of each feature in the concatenated vector with the mean raw SHAP values for the high-performing LASSO model. The feature importance was then summed within each descriptor. Atom-Pair and MAP4 were excluded because they enumerate pairs that are essentially a subset of TDiMS features. Comparing the SHAP ratios of these overlapping features does not provide meaningful insights.

Extended Data Fig. 1b presents a comparison of the proportions of the group SHAP (gSHAP) values. A gSHAP value is defined as the magnitude of the sum of the raw SHAP values derived from each descriptor with the best-performing configurations (see ‘Computation of feature importance’ section in the Methods). As expected, TDiMS features were more important for Chromophore, where the TDs of substructures played a crucial role. Supplementary Information section 2.5 describes the distribution of the SHAP values.

To further reveal the characteristics of the descriptors, we concatenated all of the descriptors except Atom-Pair and MAP4 and compared the feature importance subtotals on the basis of the descriptor type. Extended Data Fig. 1c shows the proportions of each descriptor. The features of the substructure pairs of TDiMS were utilized most for Chromophore, whereas Mordred was most important for QM9 LUMO. This finding clearly demonstrates that enumerating substructure pairs to calculate intramolecular TDs is effective for characterizing large molecules.

#### Other analyses

**Extended Data Fig. 2:**
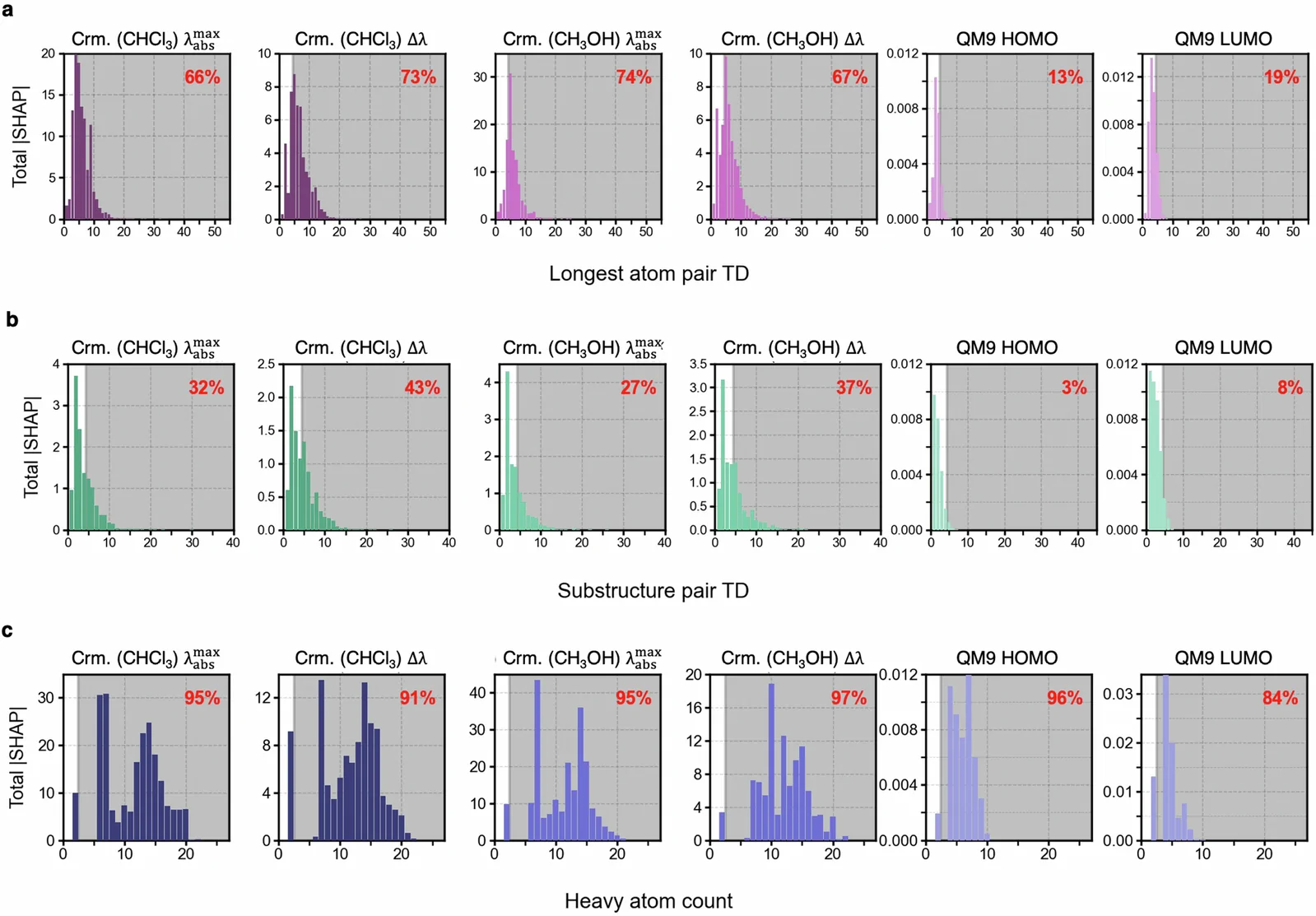
Distribution of the SHAP values for the TDiMS features. The number in red at the top right of each chart indicates the percentage of the total absolute SHAP values for the TDiMS features located in the shaded area. **a**, Categorization based on the longest topological distance (TD) between two atoms in each substructure pair. The areas with TD values of five or greater are shaded. **b**, Categorization based on the TD of each substructure pair. The areas with TD values of five or greater are shaded. **c**, Categorization based on the heavy atom count of each substructure pair. The area with a heavy atom count of two or greater is shaded. Source data.

We further investigated the factors underlying the effectiveness of TDiMS. Feature contributions of TDiMS when used alone are presented in Extended Data Fig. 2, with further discussion in Supplementary Information section 2.6. Supplementary Information section 2.8 reveals quantitative threshold of the effectiveness of TDiMS related to the molecular size in the case of dipole moment prediction using the PubChemQC dataset^17^ (see also ‘PubChemQC datasets and custom substructures of TDiMS’ section in the Methods). Finally, Supplementary Information section 2.9 analyzes the TDiMS configurations and their performance.

### Interpretation of TDiMS features in materials science

#### Insights and validity of TDiMS features

In the context of materials science, we interpret essential substructure pairs identified by TDiMS for predicting ${\lambda }_{{\rm{abs}}.}^{\max }$ and Δ*λ*. In general, these substructure pairs are closely related to photophysical processes in which molecular and electronic structures are the primary factors for determining these properties (see Supplementary Information section 3.1 for further explanations). Understanding feature contributions is necessary for the successful, effective spatial arrangement of structural units within molecules to achieve optimal light absorption.

**Extended Data Fig. 3:**
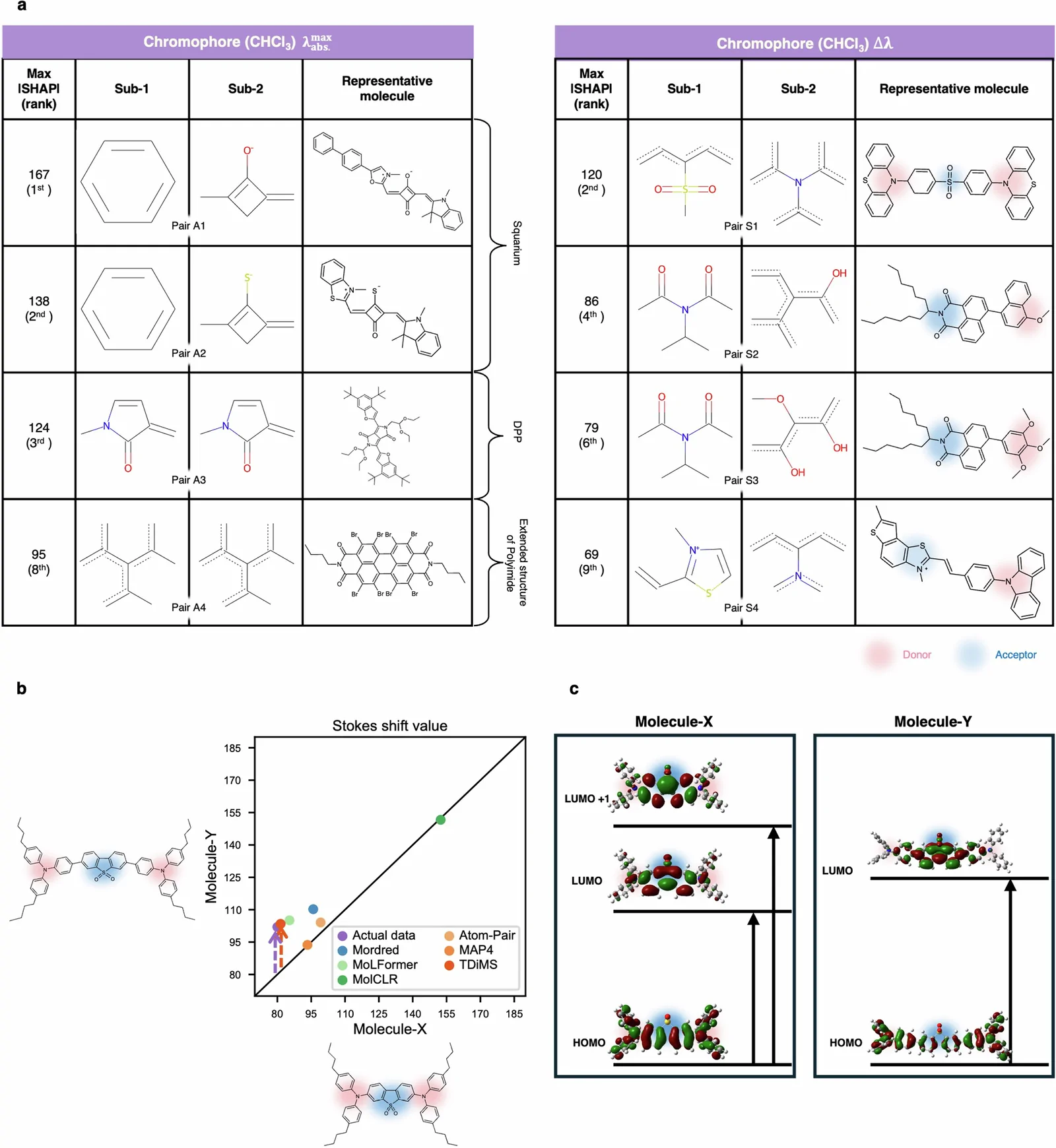
Scientific interpretation of the substructure pairs. **a**, Substructure pairs with high absolute SHAP values and representative molecules containing these pairs for chromophores with solvent CHCl_3_. **b**, Actual and predicted *Δλ* values of two structurally similar molecules with different properties. Predictions for MoLFormer and MolCLR are obtained from their fine-tuned models. **c**, Molecular orbital diagrams of Molecule-X and Molecule-Y. Atom colors are based on the GaussView default CPK colors: gray for carbon (denoted as C), white for hydrogen (H), blue for nitrogen (N), red for oxygen (O), and yellow for sulfur (S). For orbital isosurfaces, positive and negative phases + *ψ* and − *ψ* are colored in red and green, respectively. The isovalue is set to 0.02 a.u. See the Supplementary Data File for the coordinates of these two molecules. Source data.

Extended Data Fig. 3a shows representative examples of TDiMS features with large absolute SHAP values, sorted in descending order and obtained using each high-performing LASSO model for Chromophore with solvent CHCl_3_. The feature sets contributing to the predictions substantially differed between ${\lambda }_{{\rm{abs}}.}^{\max }$ and Δ*λ*, indicating that each prediction task relies on distinct features that reflect material scientific interpretation. See Supplementary Information section 3.2 for details.

#### TDiMS features and physicochemical phenomena

We finally investigated the ability of TDiMS features to capture physicochemical phenomena by analyzing their influence on property values within molecular structures.

Extended Data Fig. 3b,c shows predicted and actual Δ*λ* values for two molecules and their orbital diagrams, providing the advantages of TDiMS (also see Supplementary Information section 3.3).

**Extended Data Fig. 4:**
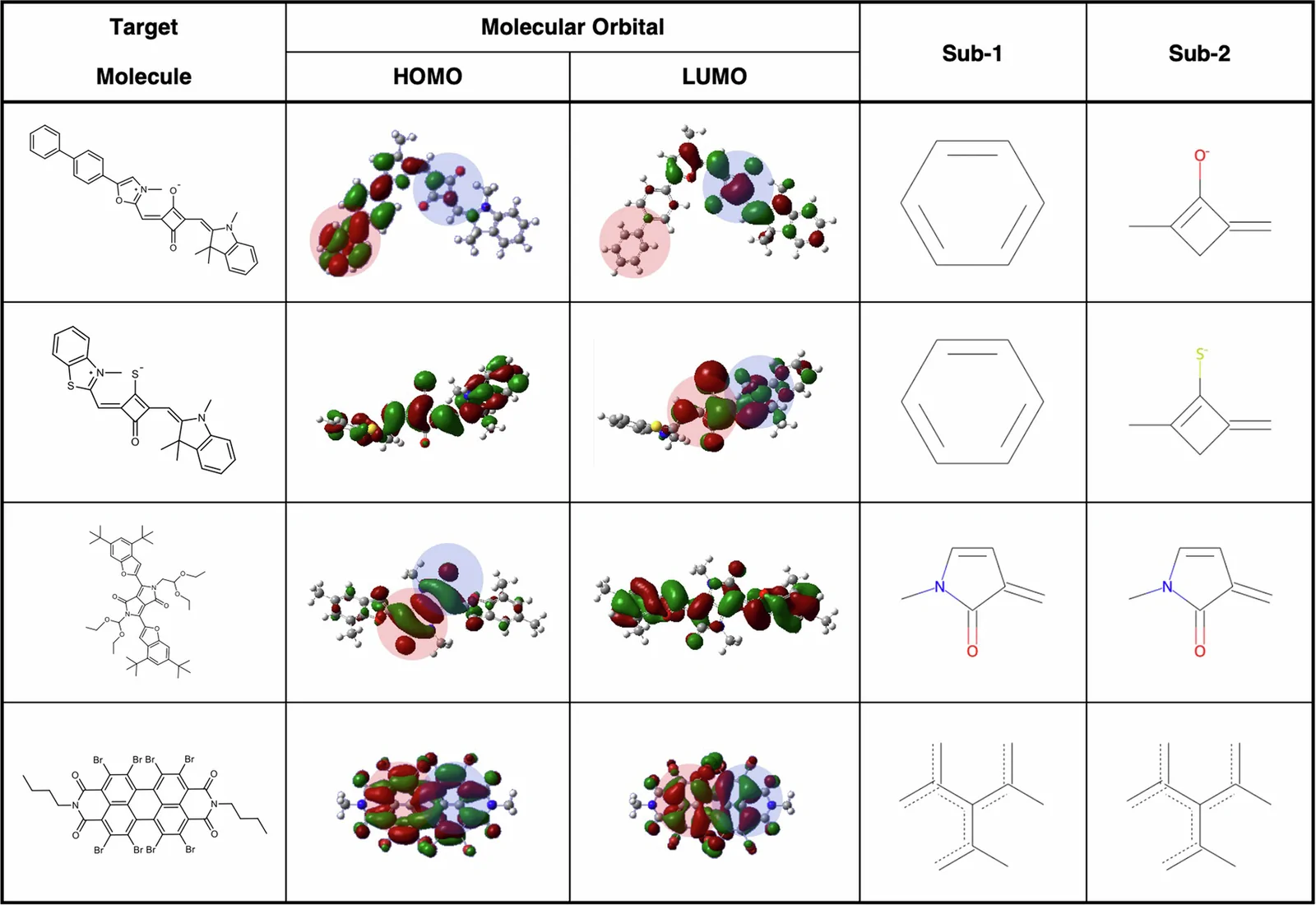
Diagrams of the molecular orbitals for the HOMO and LUMO. The DFT and TD-DFT calculations were performed for the four representative molecules shown in the ${\lambda }_{{\rm{abs}}.}^{\max }$ examples in Extended Data Fig. 3a. Atom colors are based on the GaussView default CPK colors: gray for carbon (denoted as C), white for hydrogen (H), blue for nitrogen (N), red for oxygen (O), yellow for sulfur (S), and dark red for bromine (Br). For orbital isosurfaces, positive and negative phases + *ψ* and − *ψ* are colored in red and green, respectively. The isovalue is set to 0.02 a.u. See the Supplementary Data File for the coordinates of these four molecules.

Extended Data Fig. 4 provides the results of our molecular orbital analysis based on density functional theory (DFT) and time-dependent density functional theory (TD-DFT) calculations and reveals evidence that TDiMS adequately captures the optical properties. Therefore, we conclude that, for these important examples, TDiMS successfully extracts the substructure pairs with increases or decreases in electrons, and the travel distance of the electrons between these pairs is closely related to determining the ${\lambda }_{{\rm{abs}}.}^{\max }$ values. See Supplementary Information section 3.4 for further insights.

### Limitations and future work

TDiMS addresses only two-dimensional substructures with limited stereochemistry. MolBar^18^ is a unique molecular identifier with full support of stereochemistry, using three-dimensional fragments for the global molecular topology. While MolBar can be regarded as a descriptor, its compression scheme might lose essential information for property prediction. By contrast, TDiMS enumerates all possible substructure pairs and automatically identifies essential pairs during training. Combining TDiMS with MolBar is one complementary extension that supports the analysis of diverse molecules.

TDiMS also provides numerous avenues to explore. For example, integrating TDiMS with NN-based descriptors can benefit from both approaches for further performance improvement while ensuring interpretability. Analyses involving other prediction tasks, for example, aqueous solubility and thermal stability, are important to further elucidate the behaviors of TDiMS. Establishing an approach to create a custom substructure set that fits well with the objective is necessary. Extending TDiMS to design new molecules can facilitate trial-and-error steps by experts. Finally, TDiMS is a promising candidate for addressing molecules larger than chromophores and challenging prediction tasks, for example, polymers and glass transition temperatures, for semiconductor applications.

## Methods

This section provides details about TDiMS as well as the descriptors used as baselines. In addition, the configurations for the property prediction models and the SHAP computation are described.

### TDiMS details

We use a standard definition for a molecular structure represented as a graph that consists of labeled nodes and edges without three-dimensional coordinates, where a node and an edge correspond to an atom and a bond, respectively. A graph *G*_sub_ is defined as a substructure to a molecular structure *G* if *G*_sub_ is a subgraph of *G*. *G*_sub_ is called a substructure only if *G*_sub_ is intended to be used for identifying a substructure in a molecule.

For each pair of substructures, subA and subB, the TD td_subA,subB_ is calculated as the average of the shortest bond distances across all pairs of heavy atoms in subA and subB:

1 $${\mathrm{td}}_{\mathrm{subA},\mathrm{subB}}=\frac{{\sum }_{i=1}^{{N}_{{\rm{A}}}}{\sum }_{j=1}^{{N}_{{\rm{B}}}}{\mathrm{bd}}_{{\alpha }_{i}{\beta }_{j}}}{{N}_{{\rm{A}}}\times {N}_{{\rm{B}}}},$$

where *N*_A_ and *N*_B_ are the numbers of heavy atoms in subA and subB, respectively; *α*_*i*_ and *β*_*j*_ denote the *i*th and *j*th heavy atoms in subA and subB, respectively; and ${\mathrm{bd}}_{{\alpha }_{i}{\beta }_{j}}$ is the shortest bond distance between *α*_*i*_ and *β*_*j*_. All the shortest distances are calculated using the Floyd–Warshall algorithm^19,20^ and are available as a distance matrix in RDKit (https://www.rdkit.org).

Afterward, TDiMS calculates the set of feature values TD based on the TDs:

2 $${\rm{TD}}={\{{\,f}_{\rm{dis}}(\mathrm{td}_{\rm{subA,subB}})\}}_{({\rm{subA}},{\rm{subB}})\in P},$$

where *P* is a set of all substructure pairs, and *f*_dis_(*x*) = *x*^−2^, *x*^−1^ or *x* is investigated in this study.

In the first two cases of *f*_dis_(*x*), closer distances are more influential and typical effects of materials, that is, Coulomb’s law or potential^21^, are considered, whereas in the third case, farther pairs are considered more influential, that is, the length in a conjugate system is used^22^. Depending on the target phenomena, TDiMS is extendable to support other calculation methods.

In generating substructure pairs, TDiMS currently considers the following types: (1) fragments from the Harvard Clean Energy Project (CEP)^23^ for organic solar materials, (2) circular substructures derived from the Morgan fingerprint^7^ with radii of 1 and 2 and (3) heteroatoms.

When substructures are extracted from Morgan fingerprints or fragments, smaller substructures are often fully contained by a larger superset substructure. Considering both small and large substructures causes a duplicate count of the same effect stemming from these substructures. TDiMS, therefore, eliminates pairs of smaller substructures if they are completely included in larger ones (for example, step 2, pair *k* in Fig. 1). Another source of duplication arises from identical substructure pairs appearing in multiple different locations. Their effects depend on several factors, including target molecules and physical properties. For these identical pairs, their TD values undergo a specific processing function *f*_dup_(*d*), where either the summation or the maximum is currently considered, depending on which pair should be more highly valued (for example, step 2, pair *l*).

Finally, TDiMS generates the integrated set of the substructure pairs among all the molecules in the entire dataset and represents a molecule as a feature vector that stores the TD values for those substructure pairs. The final set of enumerated substructure pairs for the dataset depends on the set of molecules in the dataset. However, TDiMS uniquely determines the substructure pairs of each molecule. When calculating a feature vector for a target molecule, a value of 0 is stored for an element of a substructure pair that is not found in that target molecule but that is available in the integrated set.

There are several possible combinations of features for TDiMS. For all combinations of configurations based on (1) the inclusion or exclusion of CEP fragments, (2) the use of circular substructures from the Morgan fingerprints with radii of *r* = 1 or 2, (3) the selection of *f*_dis_ = *x*^−2^, *x*^−1^ or *x*, and (4) *f*_dup_ = sum or max, TDiMS attempts to perform some initial training, and the most promising combination is passed to the estimator. Heteroatom features are always included. Although another possible approach is to concatenate all the cases of *f*_dis_ and *f*_dup_ and represent them as one feature vector, we do not implement this approach because of the impractical size of the feature vector.

### QM9 and Chromophore datasets

We established three datasets with various properties and molecular structure sizes: Chromophore^5^, using solvents CHCl_3_ and CH_3_OH, with absorption maxima (nm) (${\lambda }_{{\rm{abs}}.}^{\max }$) and Stokes shift (Δ*λ*) as target properties, and QM9^24,25^, with the highest occupied molecular orbital (HOMO) and lowest unoccupied molecular orbital (LUMO) values. To simulate the practical limitation of dataset size, we randomly sampled 1,000 unique molecules. Various solvents were included in the original Chromophore dataset. We chose only solvents CHCl_3_ and CH_3_OH, each of which has approximately 1,000 molecules.

Supplementary Fig. 1 shows the statistics for each dataset. While QM9 is included as a well-known benchmark, our main focus is on Chromophore because of its more complex structures, which often include many heavy atoms and rings, unlike QM9, which includes only up to nine heavy atoms. Thus, Chromophore poses challenges to descriptors, including NN-based descriptors. See Supplementary Information section 1.1 for a detailed discussion.

In preparing the 200-molecule datasets, in addition to using the same evaluation metrics as for the 1,000-molecule datasets, we generated five datasets of 200 molecules for each scenario by randomly partitioning the corresponding 1,000-molecule dataset into five disjoint subsets. This partitioning allowed us to perform five evaluations per prediction task.

### PubChemQC datasets and custom substructures of TDiMS

To create datasets for dipole-moment prediction used in Supplementary Information sections 2.7–2.9, we used molecular IDs 000000001–001539892 from the original PubChemQC database^17^ and constructed two types of dataset via random sampling: (1) a 1,000-molecule dataset regardless of heavy atom counts (HACs) and (2) five 1,000-molecule datasets grouped by HACs. For the latter datasets, the HAC intervals were set to 0–8, 9–16, 17–24, 25–32 and ≥33, where *X*–*Y* denotes the lower and upper bounds.

The settings for performance evaluation were identical to those used to obtain Table 1 except the fragment database for TDiMS to consider. Because there is no fragment database that is suitable for determining the dipole moment, we created a custom substructure set extracted from the PubChemQC datasets by the MacFrag method^26^. This custom substructure set was considered as a candidate to include or exclude features instead of CEP (see Supplementary Fig. 6 for the list of the substructures).

### Baseline descriptors

We evaluated the *R*^2^ scores of the property prediction models using five distinct baseline feature vectors derived from various representative descriptors: One knowledge-driven descriptor for comprehensive physical chemistry, two high-performing NN-based descriptors based on CLMs and GNNs, and two enumerative descriptors based on intramolecular TDs.

These descriptors are summarized as follows:

1. Mordred is a state-of-the-art open-source software capable of calculating more than 1,800 two- and three-dimensional physical/chemical features.
2. MoLFormer is a transformer-based CLM that regards SMILES as chemical language.
3. MolCLR, a state-of-the-art GNN model that can effectively handle atom connectivity, is trained via contrastive learning.
4. Atom-Pair, the shortest bond path between all pairs of atoms is extracted.
5. MAP4, a MinHash-based descriptor, is similar to Atom-Pair but targets both the atoms and the circular substructures of the Morgan fingerprint.

For MoLFormer and MolCLR, we used two settings for downstream molecular property prediction: embedding-only and fine-tuning. In the embedding-only setting, the intermediate embedding layers of the pretrained models were kept frozen and served as molecular descriptors that are learned feature vectors encoding structural and chemical information. These fixed descriptors were then passed to separate predictors trained to predict target properties. Meanwhile, in the fine-tuning setting, all parameters in both pretrained and predictor networks were updated in an end-to-end fashion. Here, as the descriptor and predictor components are no longer distinct, comparisons with other methods are based solely on predictive performance.

We describe these descriptors and their configurations in detail in this subsection.

#### Mordred

Mordred has been commonly used as an effective descriptor software, offering a total of 1,825 two- and three-dimensional descriptors, including simple atom counts, electron features and graph energies. In our evaluation, after the feature vectors were calculated via the Calculator package in Mordred (https://github.com/mordred-descriptor/mordred) in the full descriptor mode with and without the ignore_3D parameter, the feature vector yielding the best *R*^2^ score was selected. The remaining parameters were left as default settings.

#### Atom-Pair

The Atom-Pair fingerprint implemented in RDKit quantifies the occurrence of each pair of atoms, accounting for their bond distance in the target molecule. The original Atom-Pair fingerprint of RDKit returns a vector of dimension size 8,388,608, which is too large to be passed to an estimator. However, because many of the elements in the vector were zeros, the feature vector of our Atom-Pair implementation excluded the elements with all zeros throughout the dataset. Thus, the size of the feature vector was feasible for property prediction.

#### MAP4

Analogous to the Atom-Pair fingerprint, the MinHashed atom-pair fingerprint up to four bonds (MAP4) encodes atom pairs along with the corresponding bond distances. However, in MAP4, the atom characteristics are replaced by the circular substructure around each atom of the pair. Moreover, MAP4 uses the MinHashing technique of MHFP6^8^ and generates a set of unique integers *S*, representing all pairs that account for their TDs in a molecule.

MinHashing represents a feature vector whose *j*th element is calculated by the following *h*_min_ function:

3 $${h}_{\rm{min}}(S,{a}_{j},{b}_{j})={\mathop{\min }\limits_{v\in S}}((({a}_{j}v+{b}_{j})\,\,\mathrm{mod}\,\,p)\,\,\mathrm{mod}\,\,m),$$

where *p* = 2^61^ − 1 and *m* = 2^32^ − 1, *a*_*j*_ and *b*_*j*_ are precomputed random numbers.

Because each *h*_min_ call aims to randomly capture one element in *S*, MAP4 can only approximately represent *S*. The MAP4Calculator package (https://github.com/reymond-group/map4) was used to calculate a feature vector with default parameters, including a dimension of 1,024 and a radius of 2.

#### MoLFormer

The molecular language transformer (MoLFormer)^11^ is a CLM that receives a molecule as a SMILES string and is trained by a masked language framework to learn its latent representation constructed as a feature vector of 768 dimensions. A tokenizer segments a SMILES string into a sequence of tokens, where a certain percentage of tokens is masked. MoLFormer attempts to correctly reconstruct the masked sequence of tokens converted back to the original SMILES string.

An attention mechanism forms the foundation of transformer-based models, including MoLFormer, and is interpreted as a dictionary with a query, a key and a value. For a query **q**_*m*_, represented as a vector embedding the characteristics of the *m*th token, the attention mechanism calculates weights for each key **k**_*n*_, which is an embedded vector of the *n*th token. On the basis of those weights and a value **v**_*n*_ that is also an embedded vector to the *n*th token, the attention mechanism calculates a weighted value that indicates the importance of the *n*th token for the *m*th token. The final score for **q**_*m*_, calculated as a total sum of the weighted values for all the tokens, approximately indicates a relationship between one atom/bond (or a very small substructure depending on the tokenizer) and all the atoms and bonds within a molecule.

MoLFormer uses the following attention mechanism with rotary positional encoding^27^ for its NN architecture:

4 $${\mathrm{Attention}}_{m}(Q,K,V)=\frac{\phi {({R}_{m}{\mathbf{q}}_{m})}^{{\rm{T}}}\mathop{\sum }\nolimits_{n = 1}^{N}\phi ({R}_{n}{\mathbf{k}}_{n}){\mathbf{v}}_{n}}{\phi {({R}_{m}{\mathbf{q}}_{m})}^{{\rm{T}}}\mathop{\sum }\nolimits_{n = 1}^{N}\phi ({R}_{n}{\mathbf{k}}_{n})},$$

where *ϕ* is a function that returns a vector on features and *R*_*i*_ is a matrix on rotary positional encoding for the *i*th token that rotates an embedded vector.

Compared with the attention mechanism of the original transformer, this attention mechanism can substantially accelerate its training step since it calculates the summations over *n* only once and reuses them for all *m* in Attention_*m*_. In addition, *R*_*i*_ can account for the relative location of each token to improve performance. However, because *R*_*i*_ is based on a location in a sequence, an accurate bond distance between atoms in a molecular structure graph cannot be considered.

For our performance evaluation, we used the original MoLFormer implementation of Ross et al. (https://github.com/IBM/molformer) and a publicly available model trained with 10% of the molecules in the ZINC (https://zinc.docking.org/) and PubChem datasets (https://pubchem.ncbi.nlm.nih.gov/), which included 100 million molecules. We performed a preliminary evaluation to find an optimized configuration and chose ‘MolTranBertTokenizer(‘bert_vocab.txt’)’ as a tokenizer and a checkpoint of ‘N-StepCheckpoint_3_30000.ckpt’ as a pretrained model. Default values were used for the remaining parameters. For each prediction task in Table 1, we evaluated the following two standard approaches and selected the best-performing model:

- ‘Embedding only’ returns the vector of the final encoder layer of their transformer as returned in ‘outputs[’embeddings’]’ in their original source code, with the default pooling and embedding settings unless otherwise specified. This vector is passed to the input to an estimator, as is done with the other descriptors.
- ‘Fine-tuning’ follows a common fine-tuning strategy^11^ of MoLFormer, which consists of a trainable two-layer fully connected NN with a hidden dimension of 768 (that is, the dimension of the encoder embedding) with dropout (set to 0.1) and Gaussian error linear unit^28^ layers in between, generating a single output for property prediction. All the model parameters are updated.

#### MolCLR

Molecular contrastive learning of representations via GNNs (MolCLR)^12^ leverages GNNs that can learn a latent vector (that is, feature vector) of a molecule even if a training dataset consists only of molecular structures.

In MolCLR, a molecular structure is represented as a graph, where nodes correspond to atoms and edges represent bonds. The training dataset is augmented by including new molecules generated by modifying molecules in the original dataset via three strategies: masking atoms, deleting bonds and removing subgraphs. A pair of molecules is denoted as positive if these molecules originate from the same molecule. Otherwise, it is denoted as negative. Given pairs of molecules, MolCLR uses contrastive learning to train a model by distinguishing positive pairs from negative ones, guided by the InfoNCE loss function^29^.

MolCLR performs a series of message passing steps that aggregate information from neighboring nodes and incorporate it into the central node. As a generic form, given node *v*, feature ${\mathbf{h}}_{v}^{(k)}$ and aggregated feature ${\mathbf{a}}_{v}^{(k)}$ are updated in the *k*th iteration as follows:

5 $${\mathbf{a}}_{v}^{(k)}={\mathrm{AGGREGATE}}^{(k)}\left(\left\{{\mathbf{h}}_{u}^{(k-1)}:u\in N(v)\right\}\right)$$

6 $${\mathbf{h}}_{v}^{(k)}={\mathrm{COMBINE}}^{(k)}\left(\left\{{\mathbf{h}}_{v}^{(k-1)},\mathbf{a}_{v}^{(k)}\right\}\right),$$

where *N*(*v*) is a set of neighbors of *v* and ${\mathbf{h}}_{v}^{(0)}$ is initialized with atom features such as the atomic number and chirality type. In practice, these operations also encode edge features of *v*, including the bond type.

After the above operations are repeated *T* times, the feature vector **h**_*G*_ that represents the entire molecule is finally generated by the following operation:

7 $${\mathbf{h}}_{G}=\,\mathrm{READOUT}\,(\{{\mathbf{h}}_{v}^{(k)}:v\in G,0\le k\le T\}),$$

where *G* is a set of nodes in the graph.

MolCLR supports a graph convolutional network^30^ and a graph isomorphism network (GIN)^31^ to implement the AGGREGATE, COMBINE and READOUT functions. We used a public, pretrained GIN model known to perform better than the graph convolutional network does. The pretrained model was trained with 10 million unique molecules in PubChem. The dimensions of **h**_*G*_ and *T* were set to 512 and 5, respectively.

MolCLR supports two standard ways to address our prediction task, which are available via the original MolCLR implementation of Wang et al.^12^ in their GitHub repository (https://github.com/yuyangw/MolCLR):

- ‘Embedding-only’ involves passing a feature vector of MolCLR as input to an estimator, as is done with the other descriptors. The estimator receives the fixed-size embedded vector on a molecular graph obtained from the linear layer immediately after the final global pooling layer of the five-layered GIN encoder with pretrained parameters named ‘model.pth’ in their checkpoint folder on the pretrained GIN.
- ‘Fine-tuning’ involves a trainable multilayer perceptron (MLP), referred to as the MLP head, connected to the feature vector of MolCLR used by the embedding only approach. In fine-tuning, all the model parameters are trainable, but while the model parameters used to calculate the feature vector are initialized as pretrained parameters, the model parameters for the MLP head are randomly initialized. Then, the model is trained to predict property values in a supervised manner via a training dataset that consists of pairs of molecular structures and physical property values.

For each prediction task for MolCLR in Table 1, we evaluated both approaches, and the best-performing property prediction model was selected. We trained the MLP head via full-batch learning because the dataset size was sufficiently small. The default values were used for the remaining parameters.

### Prediction model

For each prediction task, we selected models (estimators) based on prior studies^13^ that demonstrated effectiveness with small datasets. Specifically, we chose the following representative models to input feature vectors and return prediction values: elastic net regression, including LASSO and Ridge regression, and random forest regression. These estimators have interpretability advantages over other estimators.

Feature selection and hyperparameter optimization (HPO) are known to yield substantial improvements in practice. In feature selection, an effective subset of features passed to the estimator is selected. HPO optimizes hyperparameters that are updated outside the training step. Therefore, during training, HPO and feature selection were performed. After training was completed, the best-performing model was chosen. Supplementary Information section 1.2 shows that feature selection effectively removes irrelevant features, reducing the feature space to a manageable size.

All the models were implemented by wrapping each estimator of the Python scikit-learn package (https://scikit-learn.org/stable/). For each model, a grid search was conducted during HPO. The hyperparameter *α* for LASSO regression, Ridge regression and elastic net regression was explored over a discrete set of {10^*k*^∣−6 ≤ *k* ≤ 1}, {10^*l*^∣−2 ≤ *l* ≤ 3}, and {10^*m*^∣−2 ≤ *m* ≤2}, respectively, where *k*, *l* and *m* are integers. A hyperparameter L1 ratio in elastic net regression was searched over the range {0.2*k*∣1 ≤ *k* ≤ 4}, where *k* is an integer. The hyperparameter min_samples_split of the random forest regression was searched within the range of [2, 3]. The SelectFromModel class in scikit-learn was incorporated for effective feature selection.

The nested cross-validation process was implemented by using the random data split method for training and testing purposes. The repeated *k*-fold class in scikit-learn was used to produce different splits in each repetition.

### Computation of feature importance

To calculate the importance of the outputs of descriptors, we used the SHAP method^15^, which ensures locally accurate additive explanations for the ML models. Thus, the outputs $f{({\mathbf{x}})}\in {\mathbb{R}}$ of any ML model based on the input feature vector ${\mathbf{x}}\in {{\mathbb{R}}}^{M}$ are partitioned into

8 $$f({\mathbf{x}})={\phi }_{0}+\mathop{\sum }\limits_{i=1}^{M}{\phi }_{i},$$

where ${\phi }_{i}\in {\mathbb{R}}\,(1\le i\le M)$ is the *i*th SHAP value that indicates the contribution of the corresponding feature element *x*_*i*_ to the model output *f*(**x**), whereas ${\phi }_{0}\in {\mathbb{R}}$ indicates the input-independent baseline output. We note that SHAP is justified as the only consistent, locally accurate additive explanation method^15^. Theoretically, the *i*th SHAP value for a model *f* and an input **x** is computed as

9 $${\phi }_{i}={\phi }_{i}(f,{\mathbf{x}}):= \sum _{S\subset [M]\setminus \{i\}}\frac{| S| !(M-| S| -1)!}{M!}\{f({\mathbf{x}}_{S\cup \{i\}})-f({\mathbf{x}}_{S})\},$$

where ${\mathbf{x}}_{S}=\{{x}_{i}\}_{i\in S}$ is the subset of feature elements in *x* with the indices restricted to *S*, [*M*] ≔ {1, …, *M*} is the set of integers from 1 to *M*, ∣*S*∣ is the cardinality of *S*, and $f({\mathbf{x}}_{S}):= {\mathbb{E}}[f({\mathbf{x}})| {\mathbf{x}}_{S}]$ is the expected output given the partial observation **x**_*S*_.

The exact computation of SHAP values is computationally challenging, especially when the total number of feature elements *M* is large. However, SHAP values are known to be efficiently computed given some assumptions for the model *f* and the feature distribution. In particular, if the model is linear and each feature element is independent of others, the calculation of the SHAP value is reduced to

10 $${\phi }_{i}(f,{\mathbf{x}})={\beta }_{i}({x}_{i}-{\mathbb{E}}[{x}_{i}]),$$

where ${\beta }_{i}\in {\mathbb{R}}$ is the *i*th coefficient of the linear model $f({\mathbf{x}})={\beta }_{0}+\mathop{\sum }\nolimits_{i = 1}^{M}{\beta }_{i}{x}_{i}$ while ${\mathbb{E}}[{x}_{i}]$ is estimated with the sample average. Because *ϕ*_*i*_(*f*, **x**) indicates the contribution of the *i*th feature element with respect to a specific input **x** (that is, the local attribution), the global attribution can be obtained by aggregating *ϕ*_*i*_(*f*, **x**) over all the feature vectors **x** ∈ *X* of interest. Specifically, we consider (1) the group-wise average absolute SHAP (gSHAP) value ∑_**x**∈*X*_∣∑_*i*∈*D*_*ϕ*_*i*_(*f*, **x**)∣/∣*X*∣ for analyzing the importance of descriptor *D* (used in Extended Data Fig. 1), (2) the average absolute SHAP value ∑_**x**∈*X*_∣*ϕ*_*i*_(*f*, **x**)∣/∣*X*∣ for analyzing the importance of feature *i* (used in Extended Data Fig. 2) and (3) the maximum absolute SHAP value ${\mathop{\max }\nolimits_{\mathbf{x}\in X}}| {\phi }_{i}(f,{\mathbf{x}})|$ in finding contributing features interpreted in the context of materials science (used in Extended Data Fig. 3).

### Reporting summary

Further information on research design is available in the Nature Portfolio Reporting Summary linked to this article.

## Supplementary information

Supplementary Information Supplementary Figs. 1–7, Discussion and Tables 1–10.

Reporting Summary

Peer Review File

Supplementary Data 1 Source data of Supplementary Fig. 1.

Supplementary Data 2 Source data of Supplementary Fig. 2.

Supplementary Data 3 Source data of Supplementary Fig. 3.

Supplementary Data 4 Source data of Supplementary Fig. 4.

Supplementary Data 5 Source data of Supplementary Fig. 5.

Supplementary Data 6 Source data of Supplementary Fig. 7.

Supplementary Data 7 The atomic coordinates of molecule X and Y shown in Extended Data Fig. 3c.

Supplementary Data 8 The atomic coordinates of the molecules in Extended Data Fig. 4.

## Source data

Source Data Extended Data Fig. 1 Statistical source data.

Source Data Extended Data Fig. 2 Statistical source data.

Source Data Extended Data Fig. 3 Statistical source data.

## Data Availability

To ensure reproducibility, the minimum datasets required to interpret, verify and extend the findings of this study are available via Zenodo at https://doi.org/10.5281/zenodo.19343345 (ref. ^32^). The deposited files contain the exact subsets of molecules and associated property values used in the Chromophore, QM9 and PubChemQC analyses. These subsets were derived from publicly available source datasets: the Chromophore dataset via Figshare (‘DB for chromophore’), QM9 from its original public release, and the CHNOPSFCl500 subset of the PubChemQC dataset from the PubChemQC repository. Atomic coordinates for the representative molecules shown in Extended Data Figs. 3 and 4, which were used in the orbital analyses, are provided in the Supplementary Data 7 and 8. Source data for Extended Data Figs. 1–3 and Supplementary Figs. 1–5 and 7 are provided with this paper.

## Code availability

The source code of the TDiMS descriptor is available via GitHub at https://github.com/IBM/materials/tree/main/models/tdims and via Zenodo at https://doi.org/10.5281/zenodo.19411156 (ref. ^33^) under the Apache License 2.0.
