# Supplementary material for: Revisiting molecular descriptors with TDiMS for interpretable intramolecular interactions based on substructure pairs
Source: Nat Comput Sci. 2026 Sep 17;6(9):945–53. doi: 10.1038/s43588-026-01036-3 (PMC13593562; doi:10.1038/s43588-026-01036-3)
Supplement: Supplementary file 1 [file 43588_2026_1036_MOESM1_ESM.pdf]

nature computational science

Supplementary information

https://doi.org/10.1038/s43588-026-01036-3

Revisiting molecular descriptors with TDiMS for interpretable intramolecular interactions based on substructure pairs

In the format provided by the authors and unedited

Table of Contents

Contents

1 Setup for physical property prediction tasks **2**

1.1 Data characteristics . . . 2

1.2 Feature selection . . . 2

2 Predictive performance **5**

2.1 Parity plot . . . 5

2.2 Detailed insight against other descriptors . . . 6

2.3 Performance using a small dataset . . . 7

2.4 Performance of NN-based descriptors under embedding-only and fine-tuned settings . . . 8

2.5 Distributions of the SHAP values . . . 9

2.6 In-depth analysis of the superiority of TDiMS features . . . 9

2.7 Predictive performance for the dipole moment . . . 11

2.8 Analysis of the heavy atom count and effectiveness of TDiMS for dipole moment prediction . . . 12

2.9 TDiMS configurations . . . 14

3 Interpretation of TDiMS-derived substructure pairs in materials science **17**

3.1 Relation between photophysical processes and molecular structures . . . 17

3.2 Essential substructure pairs and their material scientific interpretation 18

3.3 Molecular orbital diagram of Molecule-X and Molecule-Y . . . 18

3.4 Analysis of molecular orbitals involved in the absorption maxima . . . 19

1 Setup for physical property prediction tasks

1.1 Data characteristics

As shown in Supplementary Figure 1a, the chromophore for solvent $CHCl_3$ has the highest numbers of heavy atoms, rings, and aromatic rings on average, followed by the chromophore for solvent CH_3OH. QM9 has the smallest structure, with at most nine heavy atoms.

Since chromophores are related to dyes, the Chromophore dataset features a greater number of CEP fragments derived from organic solar materials. In particular, the chromophore for solvent $CHCl_3$ has the greatest number of CEP fragments, whereas QM9 contains the smallest number of these fragments. With respect to the bond path-based topological distance between the most distant atoms within a molecule, the distance between atoms in the Chromophore dataset is three times greater than that in QM9 on average. The longest distance observed in the Chromophore dataset is 60.

As displayed in Supplementary Figure 1b, the top two molecular structures in each dataset in descending order of the number of rings indicate that more rings correspond to a more complex structure with a very long SMILES sequence. In summary, compared with QM9, Chromophore contains much more complicated structures, posing challenges to descriptors, including NN-based descriptors.

The property values for each dataset are spread across various locations, indicating that ML models need to perform property predictions for molecules with diverse value ranges (see Supplementary Figure 2).

1.2 Feature selection

Supplementary Table 1 shows the number of features selected by the feature selection process for each prediction task as well as the number of original features extracted. These values are based on the first outer split of each 1000-molecule dataset associated with Table 1 in the main paper. For TDiMS, the selected substructure pair configuration that optimizes predictive performance depends on the specific task. The original features of Mordred differ across datasets because some descriptors in Mordred return invalid values for certain molecules (e.g., NaN or extreme outlier values) in the datasets, and the corresponding dimensions are removed in such cases. MolCLR and MoLFormer are not included in Supplementary Table 1 because the best MolCLR and MoLFormer scores were obtained by their fine-tuned versions in most cases, which do not include the feature selection process.

As observed in this table, TDiMS originally creates a high-dimensional feature space whose dimension size can be more than eight times larger than that of AtomPair. Many irrelevant features are successfully removed by feature selection.

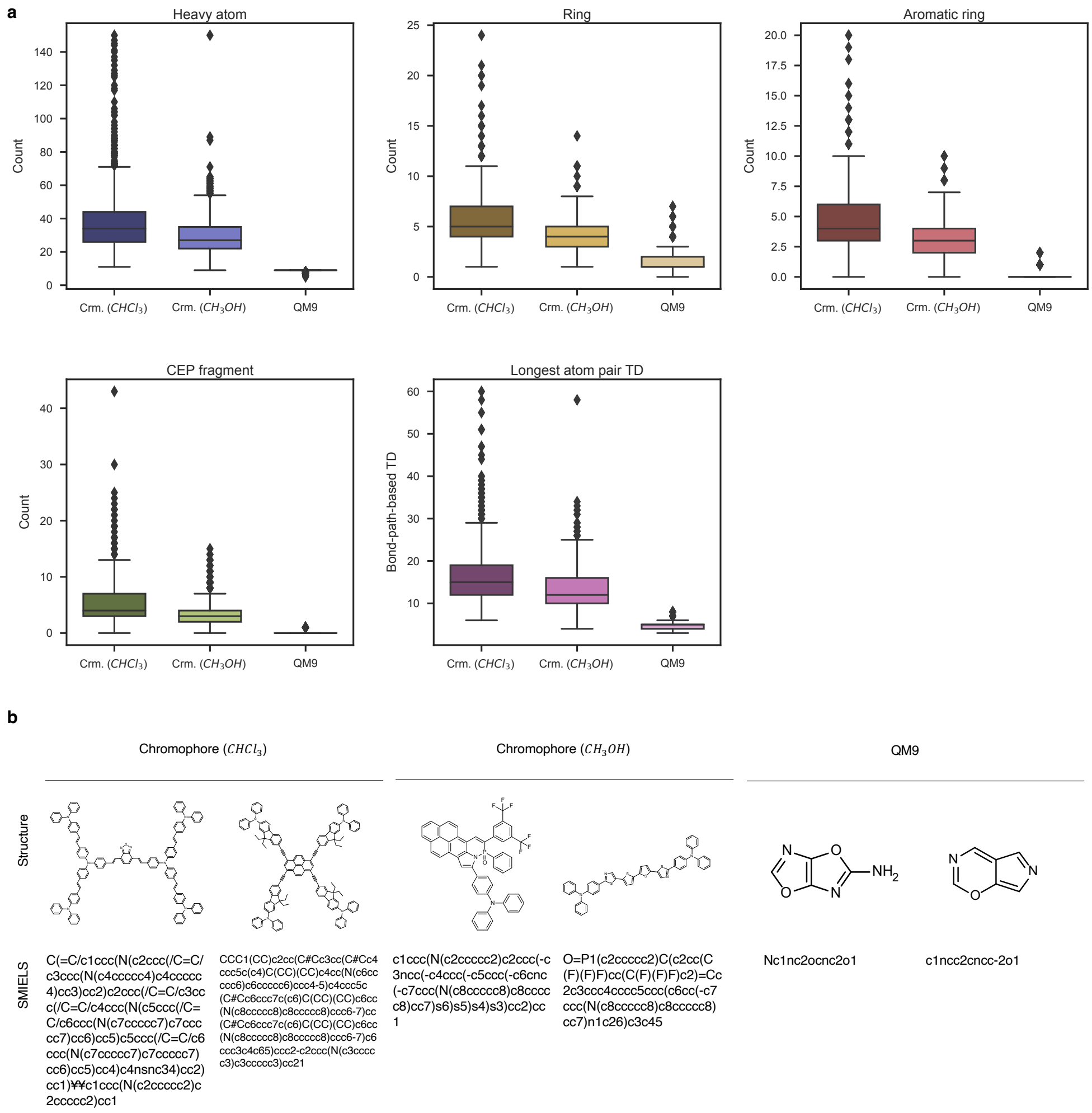

Supplementary Figure 1: **Dataset characteristics for datasets of 1000 molecules.** **a,** Box-and-whisker plots of heavy atom, ring, aromatic ring, and CEP fragment counts, and longest atom-pair topological distance (TD), for the Chromophore ($CHCl_3/CH_3OH$) and QM9 datasets. The longest atom pair TD represents the longest value of the shortest bond distance between two atoms in each molecule. Boxes, centre lines, and points indicate interquartile ranges (IQRs), medians, and outliers, respectively; whiskers extend to 1.5×IQR. **b,** The top two molecules with the largest ring counts in each dataset and their SMILES representations. Crm. stands for Chromophore, with the solvent indicated in parentheses.

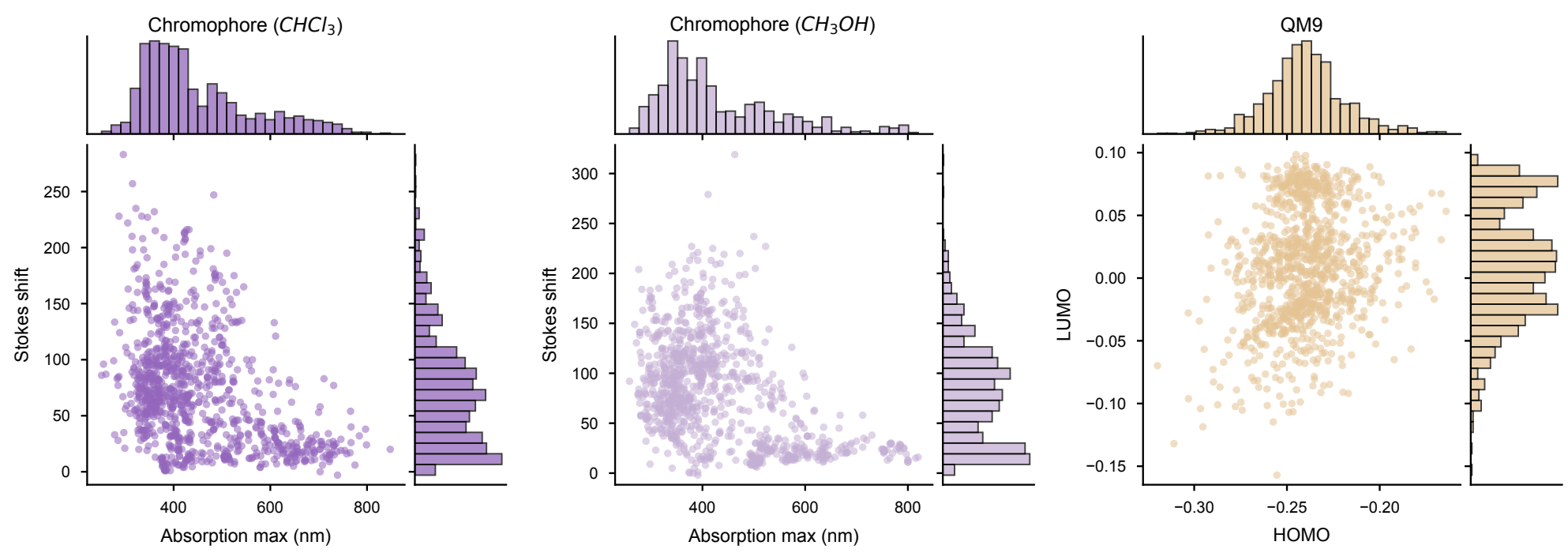

Supplementary Figure 2: **Distribution of the target property values.** Joint distributions of the two target properties for each dataset are shown as scatter plots with marginal histograms. For the Chromophore datasets, absorption maximum wavelength and Stokes shift are plotted, whereas for QM9, HOMO and LUMO energies are plotted.

Supplementary Table 1: **Dimension sizes of feature vectors for datasets of 1000 molecules.** The numbers of selected and original features are shown as "slc fs / org fs" in this table, respectively.

Descriptor	Crm. ($CHCl_3$)		Crm. (CH_3OH)		QM9	
	$\lambda_{\text{abs.}}^{\text{max}}$	$\Delta\lambda$	$\lambda_{\text{abs.}}^{\text{max}}$	$\Delta\lambda$	HOMO	LUMO
Mordred	416 / 1128	424 / 1128	400 / 1034	380 / 1034	127 / 974	132 / 974
Atom-Pair	1419 / 4522	1450 / 4522	1337 / 4284	1393 / 4284	208 / 717	221 / 717
MAP4	443 / 1024	437 / 1024	414 / 1024	427 / 1024	343 / 1024	361 / 1024
TDiMS	1858 / 40621	1439 / 40621	780 / 6387	1018 / 6387	44 / 2057	99 / 2072

2 Predictive performance

2.1 Parity plot

Supplementary Figure 3 shows parity plots of each standalone descriptor for all property prediction tasks with the best model, highlighting the characteristics of each approach.

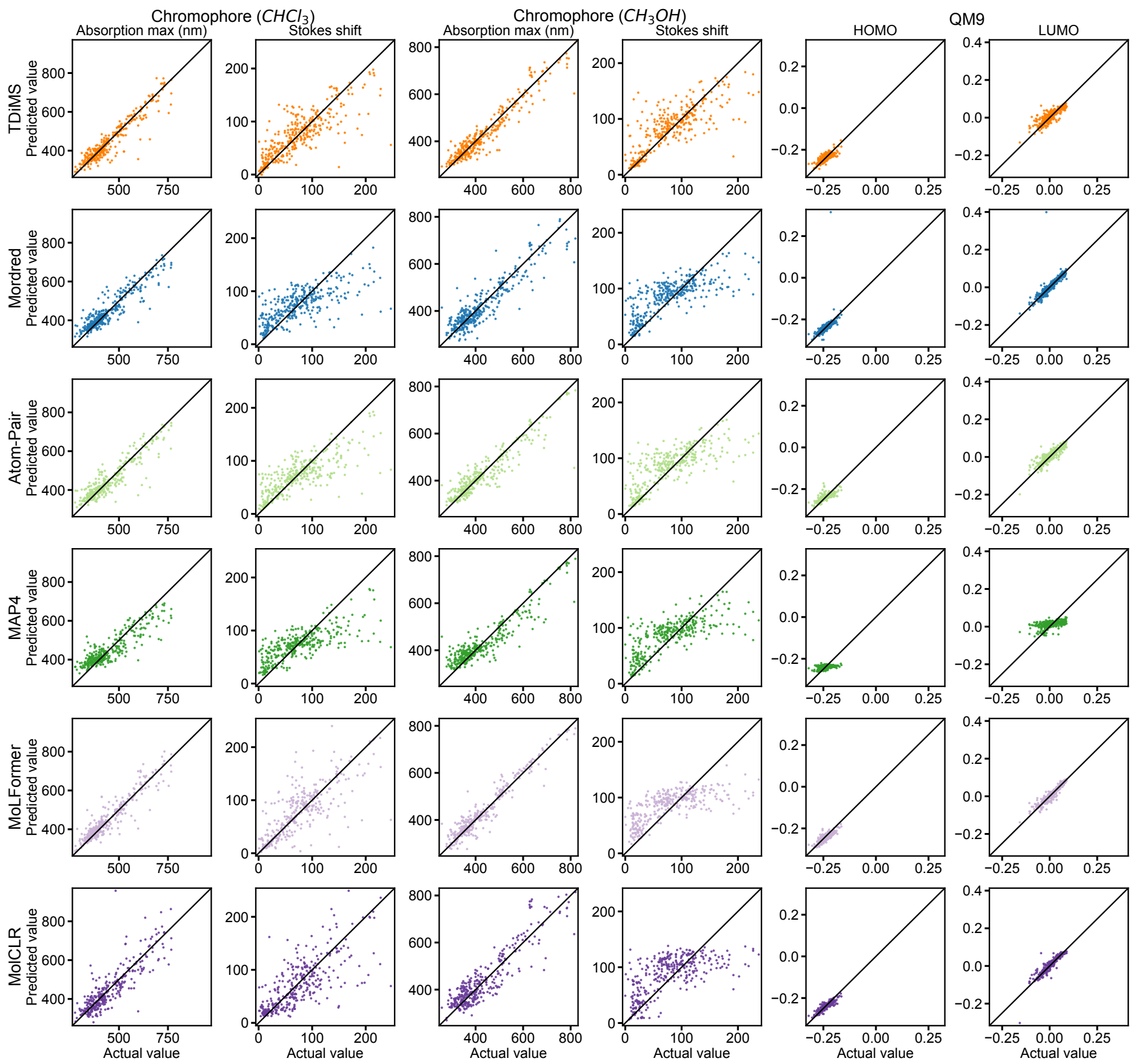

Supplementary Figure 3: **Parity plots for each descriptor across six prediction tasks.** Actual property values are plotted on the horizontal axis against the corresponding predicted values on the vertical axis. The diagonal line indicates perfect agreement. For MoLFormer and MolCLR, the plots are based on the models with the higher R^2 scores between the embedding-only and fine-tuned settings (see Supplementary Table 4).

2.2 Detailed insight against other descriptors

In relation to Table 1, we highlight comparisons against each approach from an algorithmic viewpoint.

2.2.1 Knowledge-driven descriptor

Mordred performed better than TDiMS for the HOMO and LUMO of QM9, which comprises smaller structures. This result may stem from the inclusion of many features that are not directly considered by TDiMS, for example, DetourMatrix, polarizability, Lipinski's parameters, and three-dimensional structural features. This performance aligns with the expected behaviour of TDiMS, given that QM9 provides limited opportunities for substructure pairs and TDs to exhibit their advantages. In contrast, TDiMS clearly demonstrated its strengths more effectively with the Chromophore datasets.

2.2.2 Enumerative descriptors

Atom-Pair, regarded as a special case of TDiMS, underperformed compared with TDiMS in all cases except QM9 LUMO. The superiority of TDiMS was particularly evident in the case of the Chromophore datasets. Therefore, we conclude that considering TDs across more generic substructure pairs, such as TDiMS, is essential to improve predictive performance for larger molecules.

One advantage of TDiMS over MAP4 is that TDiMS accounts for several types of substructures, including CEP fragments, when calculating the TDs. Another advantage is that TDiMS uses a more accurate representation than MAP4 does. Since MAP4 is designed for virtual screening tasks and prioritizes speed in similarity searches within large databases, it represents molecules probabilistically via MinHash values derived from locality-sensitive hashing (LSH) [1], resulting in reduced accuracy. Supplementary Table 2 highlights the superiority of TDiMS, even when it runs without CEP and uses the same circular substructures as MAP4 does.

Supplementary Table 2: **Performance comparison between MAP4 and TDiMS on the Chromophore datasets of 1000 molecules.** Values are presented as mean $R^2\pm$ SD across 30 test folds obtained from 3-fold cross-validation with 10 repeats. The same circular-substructure pairs were used for both descriptors, and the better score for each task is shown in bold.

Descriptor	Crm. ($CHCl_3$)		Crm. (CH_3OH)	
	$\lambda^{max}_{abs.}$	$\Delta\lambda$	$\lambda^{max}_{abs.}$	$\Delta\lambda$
TDiMS	**0.873±0.024**	**0.598±0.047**	**0.879±0.017**	**0.571±0.056**
MAP4	0.725±0.028	0.504±0.036	0.787±0.030	0.470±0.040

2.2.3 NN-based descriptors

Current message-passing schemes in GNNs can propagate information on atoms and their bonds only within several steps in practice [2, 3]. That is, owing to its insufficient propagation step size of five, MolCLR struggles to explicitly capture distant substructure pairs such as those located in multiple benzene rings. For example, as shown in Supplementary Figure 1, molecules in the Chromophore datasets typically exhibit bond distances exceeding 10, with the longest distance reaching 60. While MolCLR performed best for the QM9 HOMO task that comprises relatively small molecules, this observation highlights that the current step size of five in MolCLR is insufficient to directly capture such extended molecular structures in the Chromophore datasets.

Unlike GNNs, CLMs such as MoLFormer, which leverage the attention mechanism of transformer, can potentially learn relationships from one atom to other atoms and bonds, even those with long distances. However, while fine-tuning MoLFormer often achieved significant improvements, it still showed weaknesses in our evaluations. Its algorithmic drawbacks can cause performance degradation in cases with large structures, which might also be revealed as high standard deviations due to low R^2 scores of several molecules.

One drawback is that transformers can misinterpret distances between two atoms if they are part of a ring structure, as usually occurs in large molecular structures. For example, in the left molecule of Chromophore ($CHCl_3$) shown in Supplementary Figure 1, although the first and second carbons denoted as c1 in SMILES are structurally neighbours (that is, TD of 1 as a graph), their indices are 6 and 197, respectively, indicating a distance of $197 - 6 = 191$ as a sequence of characters. Additionally, because two similar molecules are often represented by very different SMILES strings, the resulting feature vectors can vary, failing to reflect the actual similarities between these two molecules [4]. While efforts have been made to address these issues [5, 6], our results with TDiMS suggest that accurately accounting for substructure pairs is an essential direction for future research.

Another challenge for transformers is their difficulty in recognizing chirality [7]. However, it is unlikely that chirality was the cause of the poorer performance of MoLFormer than TDiMS. Among the 1000 molecules in each Chromophore dataset, there are only 17 and 47 chiral molecules for solvents $CHCl_3$ and CH_3OH, respectively.

2.3 Performance using a small dataset

Supplementary Table 3 shows the R^2 scores of each descriptor when evaluated independently on the 200-molecule datasets.

All R^2 scores decreased when only a smaller dataset was available (see both Table 1 and Supplementary Table 3). However, even with the smaller dataset, TDiMS was a robust descriptor that outperformed the others in four prediction tasks with large molecular structures, that is, the Chromophore datasets. For QM9, which comprises smaller structures, the best performance was achieved by Mordred and MoLFormer (fine-tuned) for HOMO and LUMO, respectively. MolCLR was especially sensitive to changes in the dataset size against the Chromophore datasets, and its performance significantly decreased with decreasing quantity of data.

Supplementary Table 3: **Performance of each descriptor used as a standalone to perform six prediction tasks on datasets of 200 molecules.** Values are presented as mean $R^2\pm$ SD across 30 test folds, obtained from 3-fold cross-validation with two repeats on five disjoint datasets. Bold indicates the top-performing model for each task. Crm. stands for Chromophore with the type of solvent indicated in parentheses. For NN-based descriptors, the better score between embedding-only and fine-tuned settings is reported. An asterisk (*) denotes results obtained with fine-tuned settings. See Supplementary Table 5 for each setting of the NN-based descriptors.

Dataset	Crm. ($CHCl_3$)		Crm. (CH_3OH)		QM9	
Structure size	Large		Large–Medium		Small	
Property	$\lambda^{max}_{abs.}$	$\Delta\lambda$	$\lambda^{max}_{abs.}$	$\Delta\lambda$	HOMO	LUMO
MoLFormer	0.571 ± 0.116	0.259 ± 0.079*	0.682 ± 0.074	0.261 ± 0.063*	0.491 ± 0.116*	**0.800 ± 0.044***
MolCLR	0.383 ± 0.116	0.105 ± 0.118	0.420 ± 0.107	0.162 ± 0.111	0.446 ± 0.135	0.761 ± 0.049*
Mordred	0.621 ± 0.106	0.202 ± 0.101	0.676 ± 0.104	0.198 ± 0.100	**0.565 ± 0.131**	0.795 ± 0.065
Atom-Pair	0.591 ± 0.112	0.260 ± 0.064	0.631 ± 0.126	0.234 ± 0.069	0.324 ± 0.144	0.617 ± 0.087
MAP4	0.471 ± 0.097	0.149 ± 0.089	0.577 ± 0.086	0.233 ± 0.054	0.010 ± 0.072	0.094 ± 0.120
**TDiMS**	**0.633 ± 0.082**	**0.264 ± 0.091**	**0.719 ± 0.114**	**0.269 ± 0.086**	0.422 ± 0.178	0.556 ± 0.111

2.4 Performance of NN-based descriptors under embedding-only and fine-tuned settings

Supplementary Tables 4 and 5 compare the R^2 scores obtained using NN-based descriptors under embedding-only and fine-tuned settings for each prediction task on the 1000-molecule and 200-molecule datasets, respectively. While the fine-tuned settings consistently outperformed the embedding-only settings on the 1000-molecule datasets except in one case, the embedding-only settings, especially for MolCLR, became more advantageous in several tasks on the 200-molecule datasets.

Supplementary Table 4: **Performance of NN-based descriptors under embedding-only and fine-tuned settings across six prediction tasks on datasets of 1000 molecules.** Values are presented as mean $R^2\pm$ SD across 30 test folds obtained from 3-fold cross-validation with 10 repeats. Bold indicates the better score between embedding-only and fine-tuned settings for each descriptor in each task. Crm. stands for Chromophore with the type of solvent indicated in parentheses.

Dataset	Crm. ($CHCl_3$)		Crm. (CH_3OH)		QM9	
Structure size	Large		Large–Medium		Small	
Property	$\lambda^{max}_{abs.}$	$\Delta\lambda$	$\lambda^{max}_{abs.}$	$\Delta\lambda$	HOMO	LUMO
MoLFormer (embedding)	0.750 ± 0.019	0.411 ± 0.041	0.799 ± 0.019	**0.402 ± 0.054**	0.500 ± 0.050	0.682 ± 0.028
MoLFormer (fine-tuning)	**0.792 ± 0.218**	**0.500 ± 0.185**	**0.880 ± 0.066**	0.394 ± 0.139	**0.612 ± 0.044**	**0.906 ± 0.013**
MolCLR (embedding)	0.643 ± 0.040	0.404 ± 0.040	0.682 ± 0.036	0.373 ± 0.054	0.572 ± 0.033	0.696 ± 0.027
MolCLR (fine-tuning)	**0.739 ± 0.046**	**0.482 ± 0.065**	**0.781 ± 0.045**	**0.437 ± 0.065**	**0.651 ± 0.049**	**0.862 ± 0.036**

Supplementary Table 5: **Performance of NN-based descriptors under embedding-only and fine-tuned settings across six prediction tasks on datasets of 200 molecules.** Values are presented as mean $R^2\pm$ SD across 30 test folds, obtained from 3-fold cross-validation with two repeats on five disjoint datasets. Bold indicates the better score between embedding-only and fine-tuned settings for each descriptor in each task. Crm. stands for Chromophore with the type of solvent indicated in parentheses.

Dataset	Crm. ($CHCl_3$)		Crm. (CH_3OH)		QM9	
Structure size	Large		Large–Medium		Small	
Property	$\lambda^{\max}_{\text{abs.}}$	$\Delta\lambda$	$\lambda^{\max}_{\text{abs.}}$	$\Delta\lambda$	HOMO	LUMO
MoLFormer (embedding)	**0.571 ± 0.116**	0.198 ± 0.135	**0.682 ± 0.074**	0.227 ± 0.087	0.365 ± 0.129	0.587 ± 0.123
MoLFormer (fine-tuning)	0.496 ± 0.316	**0.259 ± 0.079**	0.670 ± 0.076	**0.261 ± 0.063**	**0.491 ± 0.116**	**0.800 ± 0.044**
MolCLR (embedding)	**0.383 ± 0.116**	**0.105 ± 0.118**	**0.420 ± 0.107**	**0.162 ± 0.111**	**0.446 ± 0.135**	0.544 ± 0.090
MolCLR (fine-tuning)	0.179 ± 0.090	0.071 ± 0.172	0.083 ± 0.184	0.070 ± 0.027	0.388 ± 0.166	**0.761 ± 0.049**

One possible explanation for the weaknesses of MolCLR is that its pretraining might not always learn about the structural differences of molecules because of contrastive learning based on its molecular graph augmentation strategies. For example, even if two augmented graphs generated from the same molecule have chemically different substructures, they are considered a positive example for comparison. Conversely, two identical molecules generated from two different molecules are regarded as a negative example when contrastive learning is performed. We hypothesize that fine-tuned MolCLR alleviated these potential drawbacks in its fine-tuning step with a 1000-molecule dataset.

2.5 Distributions of the SHAP values

Supplementary Figure 4 compares the SHAP values and their frequencies between TDiMS and the descriptor that achieved the best score when it was concatenated with TDiMS for each prediction task. We observed that TDiMS exhibited a more heavy-tailed distribution than Mordred or MolCLR did. As a result, compared with Mordred/MolCLR, TDiMS generated large absolute SHAP values that had a greater influence on the predicted property values (see not the frequency but the width in each graph). This phenomenon was more evident for predicting $\Delta\lambda$. On the other hand, although TDiMS also had a wider range of SHAP values than Mordred for QM9 did, these SHAP values tended to be very small and less heavy-tailed than those for Chromophore, resulting in TDiMS being unable to show its advantage.

2.6 In-depth analysis of the superiority of TDiMS features

To further investigate the factors underlying the effectiveness of TDiMS, we analysed its feature contributions when it was used alone.

Supplementary Figure 4: Comparison of the SHAP value distributions between TDiMS and the other descriptors for each prediction task. The (nongrouped) SHAP value for each feature and each molecule was plotted on the horizontal axis against the number of occurrences on the logarithmic scale vertical axis. TDiMS was compared to the descriptor that achieved the best score when concatenated in each high-performing LASSO model.

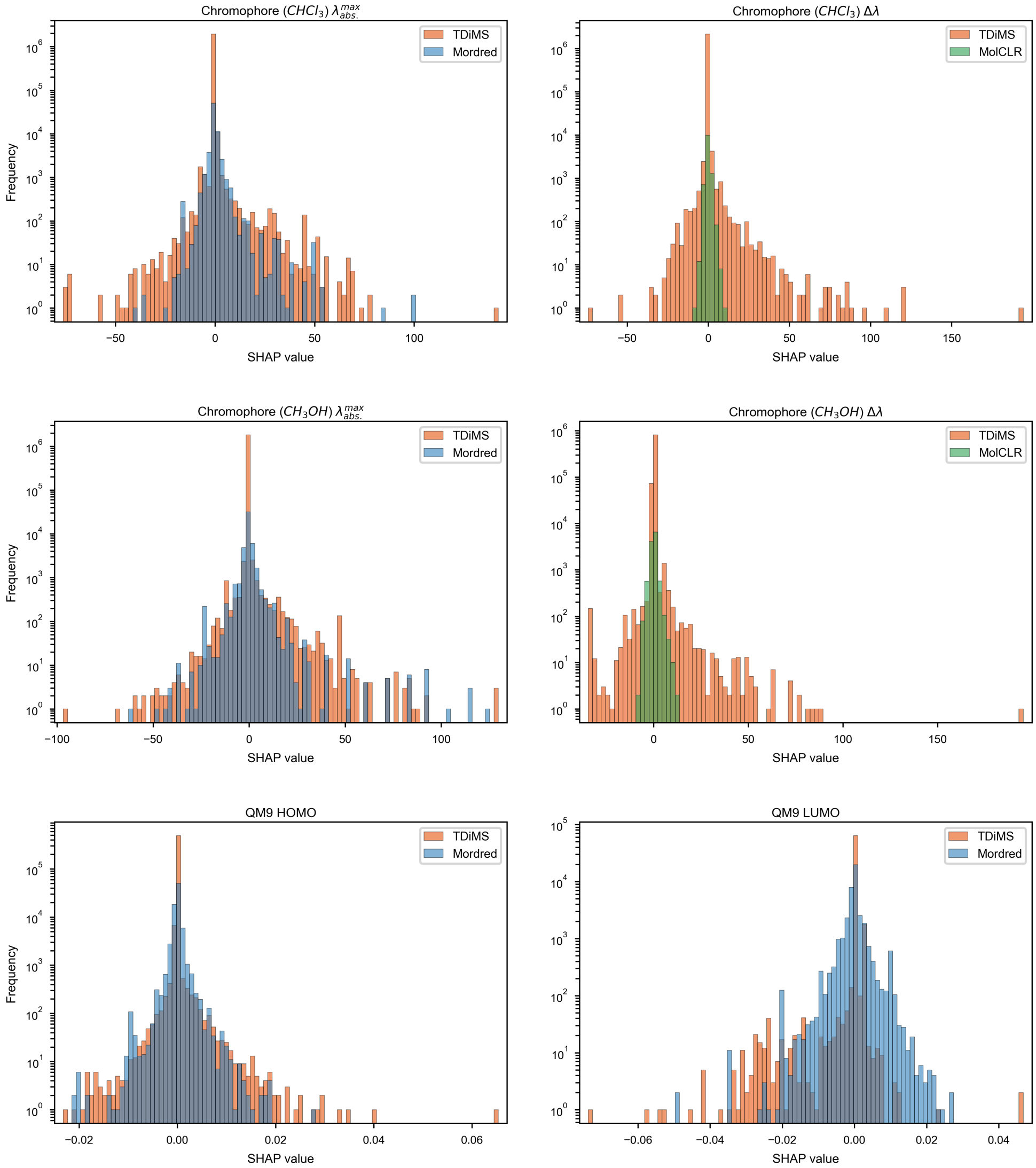

Extended Data Fig. 2a presents the total absolute SHAP values of the features categorized by the longest atom-pair bond-path-based distance for each substructure pair. Our results provide strong evidence that long-distance features effectively contribute to the predictive model, especially for Chromophore. The features with a distance of five or greater accounted for 66–74% of the predictive capability of TDiMS. See Extended Data Fig. 2b for the TDs of the substructure pairs obtained with the same procedure. This finding also implies that the current limitation to the layer size of five makes it difficult for MolCLR to efficiently capture these distant features.

Extended Data Fig. 2c shows the total absolute SHAP values of the features categorized based on the heavy atom count on each substructure pair. While Atom-Pair can capture features for only two atoms, the TDiMS features for multiple atoms were more effective for Chromophore.

2.7 Predictive performance for the dipole moment

The dipole moment of a molecule plays a fundamental role in various material properties, including the dielectric constant and interfacial characteristics of electronic materials, supramolecular assembly, and nonlinear optical responses. Rapid, accurate prediction is key for the efficient discovery of optimized molecules for high-performance polymers, organic semiconductors, and nonlinear optical materials.

Supplementary Figure 5 shows the distribution on the molecular weight and heavy atom count in our dataset. Supplementary Figure 6 shows the custom substructures.

Supplementary Table 6 shows the R^2 score of each descriptor when it was used independently. While this prediction task was difficult for all descriptors, TDiMS performed better than the others.

Supplementary Figure 5: Dataset characteristics for the PubChemQC dataset of 1000 molecules. a, Distribution of molecular weight (MolWt). **b,** Distribution of heavy atom count. In each panel, the horizontal axis indicates the property value and the vertical axis indicates frequency.

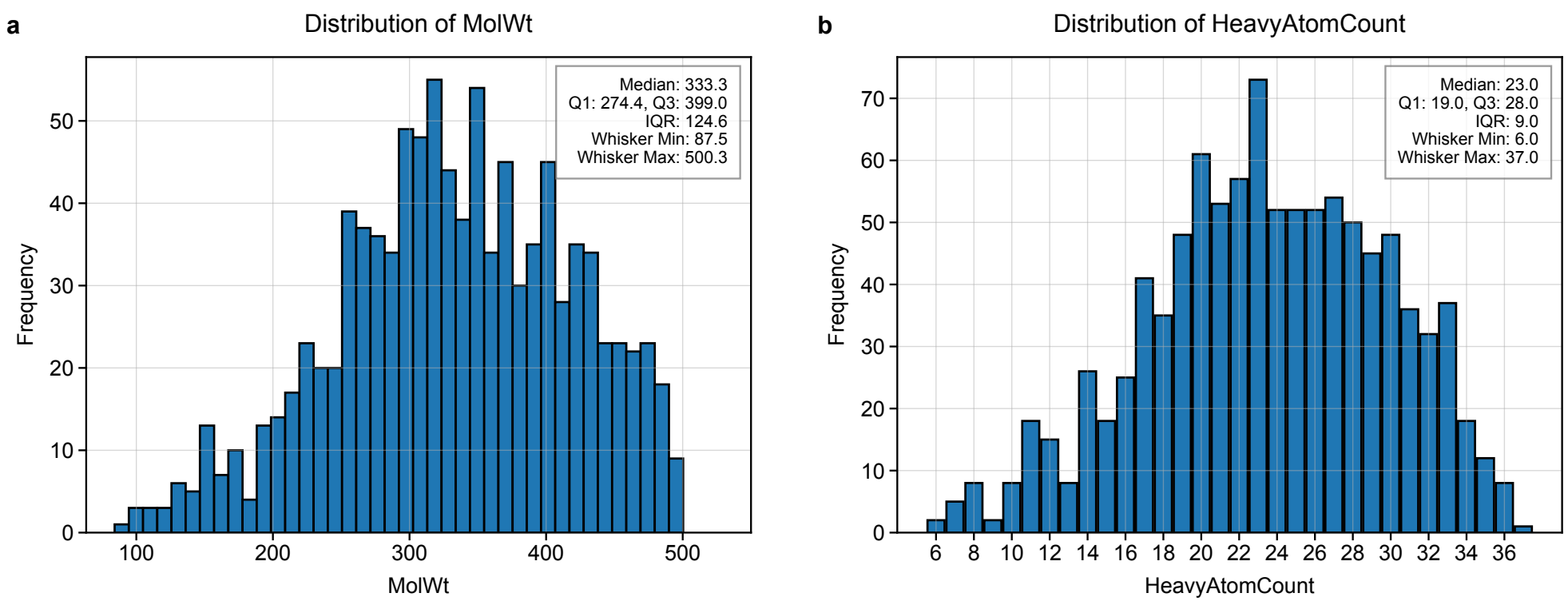

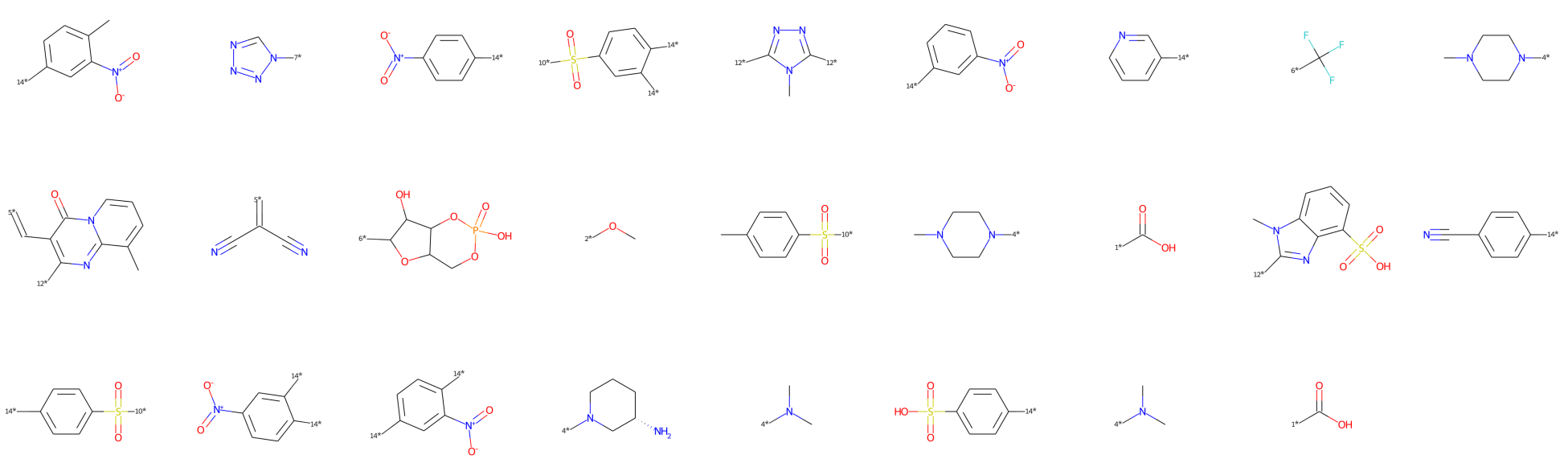

Supplementary Figure 6: **Custom substructures to test the performance of the dipole moment with PubChemQC.** Since there is no fragment database that is suitable for determining the dipole moment, we applied the MacFrag method [9] to extract these substructures from the PubChemQC datasets.

Supplementary Table 6: R^2 **score of each descriptor used independently to predict dipole moment for PubChemQC on a dataset of 1000 molecules.** Values are presented as mean $R^2 \pm$ SD across 30 test folds, obtained from 3-fold cross-validation with 10 repeats. The best score is shown in bold. The scores for MoLFormer and MolCLR are obtained from the fine-tuned models.

Mordred	MoLFormer	MolCLR	Atom-Pair	MAP4	TDiMS
0.220 ± 0.057	0.184 ± 0.048	0.134 ± 0.072	0.207 ± 0.050	0.145 ± 0.027	$\mathbf{0.235 \pm 0.039}$

2.8 Analysis of the heavy atom count and effectiveness of TDiMS for dipole moment prediction

In evaluating the performance of TDiMS with the Chromophore and QM9 datasets, we have revealed that TDiMS is effective for large molecules but displays limited performance for small molecules. One question is where a quantitative threshold lies. We attempt to answer this question in the case of dipole moment prediction using the PubChemQC dataset [8].

We first confirmed that TDiMS outperformed the others in this task (see Supplementary Section 2.7). We then created five datasets to analyse the quantitative threshold. Supplementary Figures 7a and b show the statistics on each dataset. Using these datasets, we compared TDiMS, Atom-Pair and Mordred, which were the top three descriptors in Supplementary Table 6.

Supplementary Figure 7c shows that Mordred performed best when molecules contained up to 16 heavy atoms. However, the performance difference among these three descriptors became smaller for molecules with 17–24 heavy atoms. TDiMS tended to yield better R^2 scores for the datasets with more than 24 heavy atoms, indicating that the threshold exists around this size.

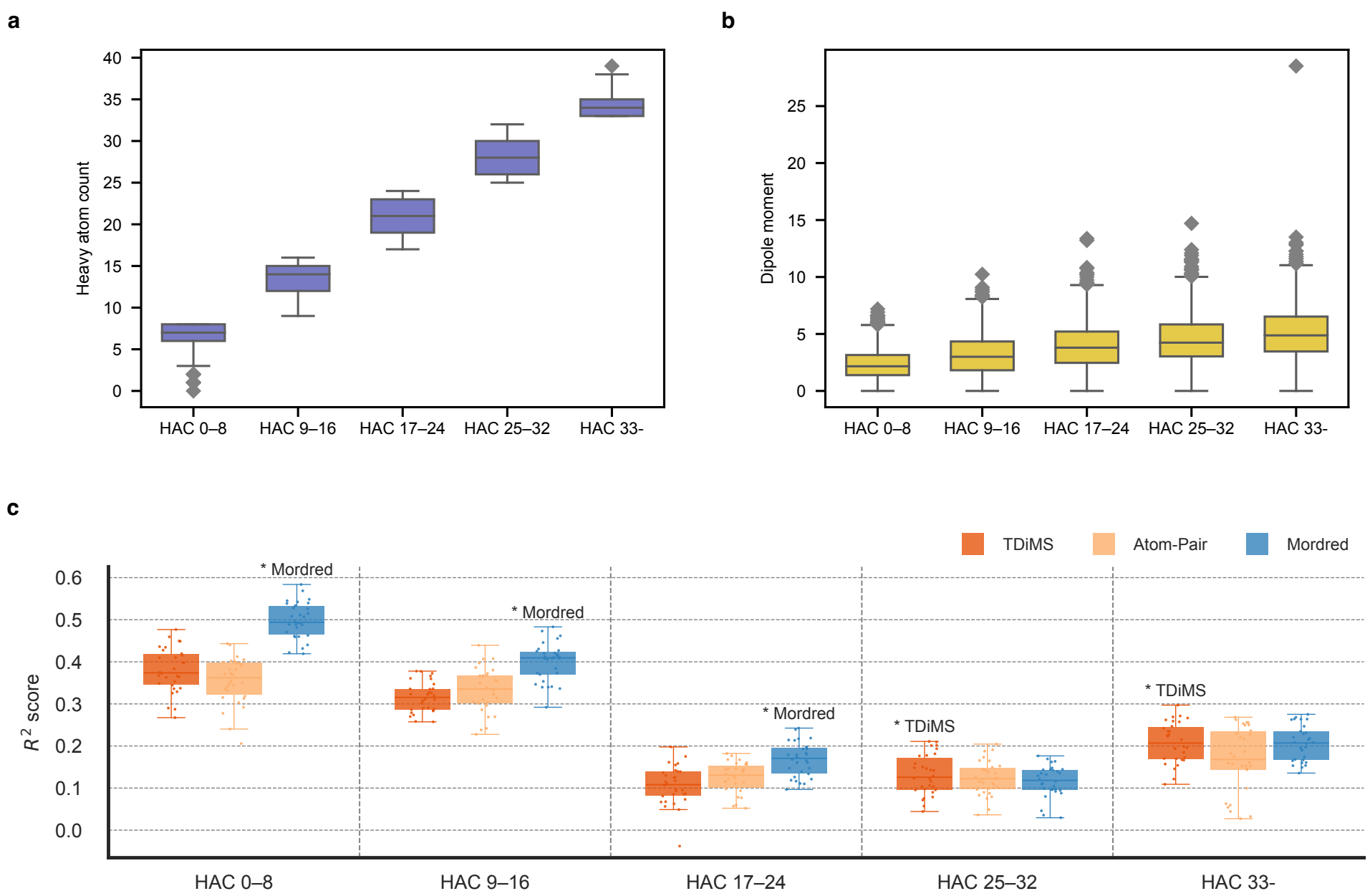

Supplementary Figure 7: Data characteristics and descriptor performance for dipole-moment prediction on PubChemQC datasets of 1000 molecules. Box-and-whisker plots for heavy-atom-count (HAC) groups. HAC X–Y and HAC 33– denote molecules with X–Y heavy atoms and at least 33 heavy atoms, respectively. **a,** Heavy atom count distributions for each HAC group. **b,** Dipole moment value distributions for each HAC group. **c,** R^2 score distributions for each descriptor across the HAC groups, calculated by 30 test folds from 3-fold cross-validation with 10 repeats. In all box-and-whisker plots, boxes, centre lines, and points indicate interquartile ranges (IQRs), medians, and outliers, respectively; whiskers extend to 1.5× IQR. In **c**, the highest-performing descriptor in each HAC group is marked with an asterisk and annotated with the descriptor name. TDiMS achieved the best performance in the HAC 25–32 and HAC 33– groups.

2.9 TDiMS configurations

We tested each of the TDiMS configurations and investigated the configuration that achieved the best score for the test dataset, which could provide additional insights for materials science. Supplementary Table 7 shows the best components for each prediction task. For the Chromophore datasets, the radius of the MFP was set to 2, indicating that larger substructures need to be considered. In contrast, the radius for QM9 was set to 1, which was attributed to its small substructures. For $\lambda_{\text{abs.}}^{\text{max}}$, the use of CEP fragments indicates the importance of considering the task-specific fragments. The x^{-1} and max operators were always the best configurations for f_{dis} and f_{dup}. However, understanding why these combinations of f_{dis} and f_{dup} were superior to the others from a materials science perspective requires reasonable formulations in physics, which is beyond the scope of this paper.

Supplementary Table 7: **Best TDiMS components.** MFP indicates the radius of Morgan fingerprint selected for extracting substructures. CEP indicates whether the CEP fragments are selected (w/) or not selected (w/o) as elements of the feature vector. $f_{dis}(x)$ indicates the method selected for calculating feature values from topological distance (x). f_{dup} indicates the operator selected for handling duplicate substructure pairs.

Method	Crm. ($CHCl_3$)		Crm. (CH_3OH)		QM9	
	$\lambda_{\text{abs.}}^{\text{max}}$	$\Delta\lambda$	$\lambda_{\text{abs.}}^{\text{max}}$	$\Delta\lambda$	HOMO	LUMO
MFP	$r=2$	$r=2$	$r=2$	$r=2$	$r=1$	$r=1$
CEP	w/	w/o	w/	w/o	w/o	w/o
$f_{dis}(x)$	x^{-1}	x^{-1}	x^{-1}	x^{-1}	x^{-1}	x^{-1}
f_{dup}	max	max	max	max	max	max

Supplementary Tables 8 and 9 show the R^2 scores when the configurations of the substructure pairs were varied. For Supplementary Table 9, the set of custom substructures shown in Supplementary Figure 6 was used.

We also observed that the choice of radius substantially affected the R^2 score. Overall, a radius of 2 was preferred for the Chromophore datasets, whereas a radius of 1 was more effective for QM9 and PubChemQC. This suggests that larger substructures were beneficial for capturing structurally extended features within chromophores, while smaller substructures that focus on localized relations were important for the prediction tasks on QM9 and PubChemQC.

Additionally, the benefit of additional substructure sets depended on the prediction task. For example, within the Chromophore datasets, incorporating CEP improved performance for $\lambda_{\text{abs.}}^{\text{max}}$, while such improvement was not observed for $\Delta\lambda$. For PubChemQC, the custom substructure list achieved substantial improvements only for the radius of 2 with HAC ≤ 16. We hypothesize that certain small substructures shown

in Supplementary Figure 6 might be able to effectively capture key features in small molecules, which were difficult to detect at the radius of 2, even though they could be identified at the radius of 1.

Supplementary Table 8: R^2 **scores of TDiMS with different Morgan fingerprint radii and with or without CEP for the Chromophore and QM9 datasets of 1000 molecules.** Values are presented as mean R^2± SD across 30 test folds obtained from 3-fold cross-validation with 10 repeats. The Morgan fingerprint radius r was set to either 1 or 2. Inclusion and exclusion of CEP are denoted by w/ and w/o, respectively. Bold indicates the best R^2 score in each task.

Method	Crm. ($CHCl_3$)		Crm. (CH_3OH)		QM9	
	$\lambda^{max}_{abs.}$	$\Delta\lambda$	$\lambda^{max}_{abs.}$	$\Delta\lambda$	HOMO	LUMO
r = 1, w/o	0.845 ± 0.020	0.554 ± 0.051	0.883 ± 0.017	0.500 ± 0.044	0.543 ± 0.043	**0.738 ± 0.029**
r = 1, w/	0.853 ± 0.020	0.581 ± 0.038	0.886 ± 0.016	0.536 ± 0.046	**0.552 ± 0.039**	0.730 ± 0.030
r = 2, w/o	0.877 ± 0.016	**0.614 ± 0.045**	0.884 ± 0.019	**0.576 ± 0.050**	0.056 ± 0.023	0.195 ± 0.030
r = 2, w/	**0.884 ± 0.016**	0.611 ± 0.045	**0.891 ± 0.016**	0.562 ± 0.052	0.143 ± 0.043	0.195 ± 0.031

Supplementary Table 9: R^2 **scores of TDiMS with different Morgan fingerprint radii and with or without the custom substructure list for PubChemQC datasets grouped by heavy atom count (HAC).** Values are presented as mean R^2± SD across 30 test folds obtained from 3-fold cross-validation with 10 repeats. The radius r was set to either 1 or 2. Inclusion and exclusion of the custom substructure list are denoted by w/ and w/o, respectively. Bold indicates the best R^2 score in each HAC group.

Method	PubChemQC (dipole moment)				
	$HAC \leq 8$	$9 \leq HAC \leq 16$	$17 \leq HAC \leq 24$	$25 \leq HAC \leq 32$	$33 \leq HAC$
r = 1, w/	0.311 ± 0.038	0.313 ± 0.038	0.093 ± 0.039	**0.132 ± 0.031**	**0.220 ± 0.040**
r = 1, w/o	**0.316 ± 0.042**	**0.332 ± 0.036**	**0.101 ± 0.032**	**0.132 ± 0.030**	**0.220 ± 0.044**
r = 2, w/	0.257 ± 0.036	0.244 ± 0.043	0.091 ± 0.035	0.111 ± 0.040	0.211 ± 0.038
r = 2, w/o	0.227 ± 0.035	0.199 ± 0.041	0.083 ± 0.036	0.112 ± 0.029	0.210 ± 0.047

Supplementary Table 10 compares the performances of TDiMS with different substructure definitions for molecules with heavy atom count (HAC) ≥ 33. The results indicate that substructure definition substantially influences model performance. Specifically, while the models using the custom substructure list performed comparably to the best models that were without fragments, both CEP and BRICS yielded lower scores. As BRICS is designed for drug-like molecules and CEP was derived from organic solar materials, neither may be well-suited for dipole moment prediction in PubChemQC. We, therefore, conclude that incorporating appropriate substructures tailored to the underlying dataset and target prediction task plays an essential role.

On the other hand, automatically extracting suitable custom substructures from the target dataset remains an important future extension to TDiMS.

Supplementary Table 10: R^2 **scores of TDiMS with different substructure definitions for the PubChemQC dataset of 1000 molecules with HAC** ≥ 33**.** Values are presented as mean $R^2 \pm$ SD across 30 test folds obtained from 3-fold cross-validation with 10 repeats. The radius r was set to either 1 or 2. The comparisons include the case without any custom substructures (w/o fragment) as well as cases with the custom substructure list, CEP, and BRICS [10]. Bold indicates the best R^2 score.

	w/o fragment	w/ custom substructure list	w/ CEP	w/ BRICS
r = 1	**0.220 ± 0.044**	**0.220 ± 0.040**	0.216 ± 0.048	0.174 ± 0.042
r = 2	0.210 ± 0.047	0.211 ± 0.038	0.202 ± 0.044	0.190 ± 0.044

3 Interpretation of TDiMS-derived substructure pairs in materials science

3.1 Relation between photophysical processes and molecular structures

This subsection provides an overview for those who are not familiar with the physical processes of molecules.

In photophysical processes, molecular and electronic structures are primary factors for determining maximum absorption wavelengths and Stokes shifts. When a molecule absorbs light, an electron within that molecule transitions from the ground state to a molecule-specific excited electronic state because of the photon energy. This transition typically occurs as a transition from the ground state (S0) to the first excited state (S1). The transition with the highest energy transition probability corresponds to the maximum absorption wavelength, that is, the absorption maxima, and its value is observed as the maximum absorption peak in the absorption spectrum of the molecule.

For typical organic molecules, this peak value corresponds to the case where an electron transitions from an occupied orbital near the HOMO to an unoccupied orbital near the LUMO. These orbital energy levels, which are determined by the stability of the electron within the molecule, are closely related to molecular design. For example, extending the length of the conjugation system can distribute π electrons over a wider area, leading to a reduction in the energy level difference. As a result, the maximum absorption wavelength is shifted towards a longer wavelength [11]. Therefore, understanding the relationships between molecular structures and their absorption wavelengths has been essential for successfully generating molecular structures of interest, such as a systematic approach to introduce electron-donating or electron-withdrawing functional groups that can adjust energy levels and control absorption wavelengths [12].

After excitation through light absorption, the molecule undergoes vibrational relaxation, transitioning to a more stable vibrational state than the current excited state. It then emits energy as fluorescence and returns to the ground state. The Stokes shift is defined as the difference between this fluorescence and the absorption energy and corresponds to the stabilization energy of the molecule in its excited state. This energy stabilization is substantially influenced by changes in the internal structure of the molecule as well as intermolecular interactions. In particular, if a molecule is polarized in polar solvents, reorganization of the surrounding solvent molecules can lead to energy stabilization. The appropriate selection and spatial arrangement of electron-rich units (donors) and electron-deficient units (acceptors) are crucial for inducing polarization within a molecule. As investigated in numerous studies, the effects of the Stokes shift are observed when combinations of these units and their distances are varied [13, 14]. The effects of different combinations of these units and their distances are reflected in the Stokes shift.

3.2 Essential substructure pairs and their material scientific interpretation

The molecules in Extended Data Fig. 3a for $\lambda_{\mathrm{abs.}}^{\mathrm{max}}$ demonstrate that TDiMS successfully identifies substructure pairs relevant for dye-like structures and conjugated units that influence $\lambda_{\mathrm{abs.}}^{\mathrm{max}}$. Moreover, these molecules, with pairs A1, A2 and A3, referred to as Squaryrium and Diketo–Pyrrolo–Pyrrole (DPP), are well known for their absorption in the long-wavelength region. A molecule with A4 has an extended polyimide structure and is often used as a fluorescent molecule. On the other hand, the substructure pairs for $\Delta\lambda$ reveal that TDiMS identified the presence of the donor and acceptor areas highlighted in red and blue, respectively, in the figure. Their spatial independence determines $\Delta\lambda$, and TDiMS successfully extracts these essential features, even in cases with a small dataset.

3.3 Molecular orbital diagram of Molecule-X and Molecule-Y

Extended Data Fig. 3b shows two molecules in the Chromophore dataset with $CHCl_3$ as the solvent and their actual and predicted $\Delta\lambda$ values. The predicted values were calculated via six descriptors, including TDiMS. The two molecules (Molecule-X and Molecule-Y) share similar structures. The essential difference between these molecules is the TD of the highlighted substructure pair S1, which is identified as an important feature in Extended Data Fig. 3a. Despite their structural similarity, the molecules exhibit distinct functions, as reflected by a 27% higher actual $\Delta\lambda$ value for Molecule-Y than for Molecule-X (see the purple arrow in the graph). TDiMS identified S1 as a major contributor in both molecules, and the predicted $\Delta\lambda$ value was successfully increased (see the dark orange arrow in the graph) as the TD of S1 increased. The increase associated with the prediction of TDiMS closely matched the actual data. MoLFormer also captured that increase but does not possess interpretability. Extended Data Fig. 3c demonstrates that the position of S1 captures the physical phenomena from the perspective of the molecular orbitals involved in electronic transitions.

Pair S1 in Extended Data Fig. 3a helps material scientists understand the different behaviors of these molecules from the viewpoint of the molecular orbitals involved in the main electronic transitions. Extended Data Fig. 3c visualizes intramolecular interactions to provide a deeper understanding of the physical phenomena associated with Molecule-X and Molecule-Y. The localization of electrons at different positions was observed in orbitals that contributed to the ground state (that is, HOMO) as well as those in the excited state (that is, LUMO). This observation implies that these molecules have electron-rich (donor) and electron-deficient (acceptor) sites within each of the molecules, and depending on their distance, intramolecular dipole moments are formed. The locations of both the donors and acceptors match those of pair S1 extracted by TDiMS as a substantial feature, leading to accurate property prediction. Interdependently, TDiMS provides chemical insight into crucial substructure pairs whose distances need to be adjusted when a new molecule is designed.

3.4 Analysis of molecular orbitals involved in the absorption maxima

We performed DFT and TD-DFT calculations for the four molecules selected in Extended Data Fig. 3a and investigated the molecular orbitals involved in $\lambda_{\text{abs.}}^{\text{max}}$. Our results shown in Extended Data Fig. 4 confirm that the HOMO–LUMO transitions were the main factors contributing to the absorption maxima in all four of these cases. Additionally, the patterns of electronic transitions varied depending on the molecular structure. Overall, TDiMS successfully identified these different patterns.

For the first molecule shown at the top of Extended Data Fig. 4, transitions occurred from one localized molecular orbital to another localized orbital, and TDiMS extracted appropriate pairs of the localized substructures involved in the electron transfer. For the second and third molecules, transitions occurred from a delocalized molecular orbital over the entire molecule to a localized molecular orbital. In this case, TDiMS captured the substructures corresponding to the regions of the transitions from the delocalized orbital to the localized orbital. Finally, for the fourth molecule at the bottom of the figure, transitions were observed from a delocalized molecular orbital to another delocalized orbital. TDiMS broadly identified the substructures where the delocalized molecular orbitals were distributed. These results demonstrate that TDiMS is capable of flexibly capturing the substructures involved in different optical transition modes depending on the molecular structure.

Our results for the first molecule in Extended Data Fig. 4 also show that the main electronic excitation corresponding to the absorption maximum involved a strong intramolecular charge transfer (ICT) transition that is typical for the donor–acceptor-type molecules. In this ICT transition, the transfer of the electron density occurred from the donor moiety to the acceptor moiety, leading to a change in the electron density distribution of the molecular orbitals. TDiMS successfully extracted the substructure pairs located in the regions where the electron density associated with such ICT processes increased or decreased.

References

[1] M., B., T., C. & P., G. Ellis, A. & Hagino, T. (eds) *LSH forest: Self-tuning indexes for similarity search.* (eds Ellis, A. & Hagino, T.) *Proceedings of the 14th International Conference on World Wide Web*, 651–660 (2005).

[2] Li, L. Q., Han, Z. & Wu, X. McIlraith, S. A. & Weinberger, K. Q. (eds) *Deeper insights into graph convolutional networks for semi-supervised learning.* (eds McIlraith, S. A. & Weinberger, K. Q.) *Proceedings of the 32nd AAAI Conference on Artificial Intelligence*, 3538–3545 (2018).

[3] Xu, K., Hu, W., Leskovec, J. & Jegelka, S. Sainath, T., Rush, A., Levine, S., Livescu, K. & Mohamed, S. (eds) *How powerful are graph neural networks?* (eds Sainath, T., Rush, A., Levine, S., Livescu, K. & Mohamed, S.) *Proceedings of the 7th International Conference on Learning Representations* (2019).

[4] Jin, W., Barzilay, R. & Jaakkola, T. Dy, J. & Krause, A. (eds) *Junction tree variational autoencoder for molecular graph generation.* (eds Dy, J. & Krause, A.) *Proceedings of the 35th International Conference on Machine Learning*, 2328–2337 (2018).

[5] Faez, F., Ommi, Y., Baghshah, M. S. & Rabiee, H. R. Deep graph generators: A survey. *IEEE Access* **0**, 106675–106702 (2021). URL https://ieeexplore.ieee.org/document/9490655.

[6] Xia, J., Zhu, Y., Du, Y. & Li, S. Z. Elkind, E. (ed.) *A systematic survey of chemical pre-trained models.* (ed.Elkind, E.) *Proceedings of the 32nd International Joint Conference on Artificial Intelligence*, 6787–6795 (2023).

[7] Yoshikai, Y., Mizuno, T., Nemoto, S. & Kusuhara, H. Difficulty in chirality recognition for transformer architectures learning chemical structures from string representations. *Nature Communications* **15**, 1–12 (2024). URL https://doi.org/10.1038/s41467-024-45102-8.

[8] Nakata, M. & Shimazaki, T. PubChemQC project: A large-scale first-principles electronic structure database for data-driven chemistry. *J. Chem. Inf. Model* **57**, 1300–1308 (2017).

[9] Diao, Y., Hu, F., Shen, Z. & Li, H. MacFrag: Segmenting large-scale molecules to obtain diverse fragments with high qualities. *Bioinformatics* **39** (2023).

[10] Degen, J., Wegscheid-Gerlach, C., Zaliani, A. & Rarey, M. On the art of compiling and using 'drug-like' chemical fragment spaces. *ChemMedChem* **3**, 1503–1507 (2008). URL https://doi.org/10.1002/cmdc.200800178.

[11] Padilha, L. A. *et al.* Nonlinear absorption in a series of Donor-p-Acceptor cyanines with different conjugation lengths. *Journal of Materials Chemistry* **19**, 7503–7513

(2009).

[12] Gouterman, M. Study of the effects of substitution on the absorption spectra of porphin. *The Journal of Chemical Physics* **30**, 1139–1161 (1959).

[13] Czaplińska, B. *et al.* Theoretical and experimental investigations of large stokes shift fluorophores based on a quinoline scaffold. *Molecules* **25** (2020).

[14] Ahmed, R. & Manna, A. K. Understanding high fluorescence quantum yield and simultaneous large stokes shift in phenyl bridged donor-π-acceptor dyads with varied bridge lengths in polar solvents. *The Journal of Physical Chemistry A* **126**, 4221–4229 (2022).
