# Supplementary material for: Revisiting molecular descriptors with TDiMS for interpretable intramolecular interactions based on substructure pairs
Source: Nat Comput Sci. 2026 Sep 17;6(9):945–53. doi: 10.1038/s43588-026-01036-3 (PMC13593562; doi:10.1038/s43588-026-01036-3)
Supplement: Supplementary file 3 [file 43588_2026_1036_MOESM3_ESM.pdf]

nature portfolio

Peer Review File

Revisiting Molecular Descriptors with TDiMS for Interpretable Intramolecular Interactions Based on Substructure Pairs

Corresponding Author: Dr Lisa Hamada

This file contains all editorial decision letters in order by version, followed by all author rebuttals in order by version.

Version 0:

Decision Letter:

** Please ensure you delete the link to your author homepage in this e-mail if you wish to forward it to your co-authors. **

Dear Dr Kishimoto,

I am forwarding this email to you, as I received an out-of-office message from Dr. Hamada.

Your manuscript "Revisiting Molecular Descriptors with TDiMS for Interpretable Intramolecular Interactions Based on Substructure Pairs" has now been seen by 2 referees, whose comments are appended below. You will see that while they find your work of interest, they have raised points that need to be addressed before we can make a decision on publication.

The referees' reports seem to be quite clear. Naturally, we will need you to address *all* of the points raised.

While we ask you to address all of the points raised, the following points need to be substantially worked on:

1) Please be sure to demonstrate and discuss the interpretability and generalizability of your approach

2) Please be sure to discuss the limitations of your approach

3) Please demonstrate and discuss the differences between TDiMS and MolBAR

Please use the following link to submit your revised manuscript and a point-by-point response to the referees' comments (which should be in a separate document to any cover letter):

Link Redacted

** This url links to your confidential homepage and associated information about manuscripts you may have submitted or be reviewing for us. If you wish to forward this e-mail to co-authors, please delete this link to your homepage first. **

To aid in the review process, we would appreciate it if you could also provide a copy of your manuscript files that indicates your revisions by making use of Track Changes or similar mark-up tools. Please also ensure that all correspondence is marked with your Nature Computational Science reference number in the subject line.

In addition, please make sure to upload a Word Document or LaTeX version of your text, to assist us in the editorial stage.

To improve transparency in authorship, we request that all authors identified as 'corresponding author' on published papers create and link their Open Researcher and Contributor Identifier (ORCID) with their account on the Manuscript Tracking System (MTS), prior to acceptance. ORCID helps the scientific community achieve unambiguous attribution of all scholarly contributions. You can create and link your ORCID from the home page of the MTS by clicking on 'Modify my Springer Nature account'. For more information please visit please visit www.springernature.com/orcid.

We hope to receive your revised paper within three weeks. If you cannot send it within this time, please let us know.

We look forward to hearing from you soon.

Best regards,

Kaitlin McCardle, PhD
Senior Editor
Nature Computational Science

Reviewers comments:

Reviewer #1 (Remarks to the Author):

Review

Manuscript: Revisiting Molecular Descriptors with TDiMS for Interpretable Intramolecular Interactions Based on Substructure Pairs

Authors: Lisa Hamada, Akihiro Kishimoto, Kohei Miyaguchi, Masataka Hirose, Junta Fuchiwaki, Indra Priyadarsini, Seiji Takeda

The authors introduced TDiMS (Topological Distance of intramolecular Molecular Substructures), a novel molecular descriptor that captures nonlocal intramolecular interactions while maintaining interpretability. Unlike conventional descriptors that either focus too locally or lack physical meaning, TDiMS enumerates substructure pairs and summarizes their topological distances, allowing for accurate prediction of molecular properties, particularly in large, complex molecules. Through evaluations on datasets like Chromophore (large organic chromophores) and QM9 (small molecules), TDiMS consistently outperformed existing descriptors, such as Mordred, Atom-Pair, MAP4, MolCLR, and MoLFormer, especially in predicting properties like absorption maxima and Stokes shifts. Importantly, feature contribution analyses using SHAP values confirmed that long-range substructure interactions were critical for predictive performance, offering insights closely aligned with known photophysical principles.

TDiMS outperforms other descriptors as shown in Table 1. However, the authors also highlight the interpretability of TDiMS in the manuscript. The interpretability of TDiMS is unclear when TDiMS is applied to predict absorption maximum, and HOMO and LUMO energies as commented below.
- Absorption maximum is more related to how the electron is delocalized in the molecule and how the electron density is changed upon light absorption. It is unclear how TDiMS can be used to understand absorption maximum. In other words, it is unclear why nonlocal intramolecular interactions in large molecules is important to accurately predict the absorption maximum of organic compounds.
- In the manuscript, substructures in a given molecule seem to play a critical role in predicting molecular properties. For a molecule, substructures can be defined in many different ways. It is unclear how the substructures can be defined in given molecules.
- For a given molecule, are the substructures the same for prediction of absorption maximum, and HOMO and LUMO energies?
- For different property prediction, the size (range) of substructures needs to be properly defined. Depending on the molecular property prediction, it is unclear whether the size (range) of substructures can be varied or not? For example, for prediction of aqueous solubility, optical properties, PLQY, and so on, are the substructures the same or different?
- The optical properties (absorption max) of organic compounds are determined by the frontier orbitals (HOMO and LUMO), which can be localized in certain substructures or delocalized in entire molecules depending on the molecular structures. It is unclear how TDiMS is related to the optical properties.
- Absorption by large molecules (donor-acceptor types) induces strong intramolecular charge transfer (ICT) within the molecules. It is unclear how TDiMS can be used to explain the ICT.
- To predict HOMO and LUMO energies of small molecules (QM9 dataset), how the substructures are defined? How the predicted HOMO and LUMO energies are varied depending on the size (range) of substructures?
- Can TDiMS be applicable to predict other molecular properties excluding optical properties and HOMO and LUMO energies, such as aqueous solubility, logP, glass temperature of polymers, and so on?
- The authors should provide a more detailed discussion or quantitative thresholds for when TDiMS is no longer advantageous (e.g., heavy atom counts, molecular weight limits).
- TDiMS relies heavily on the choice of substructure (CEP fragments, Morgan fingerprints) The method’s sensitivity to substructure selection is not fully explored. Would alternative fragmentations (e.g., random, functional groups) lead to similar or worse performance? Robustness to this choice should be discussed.
- The authors claimed that TDiMS captures photophysical phenomena (e.g., donor–acceptor distance, conjugation length). However, it is unclear whether TDiMS generalizes equally well to other types of properties (e.g., mechanical strength, thermal stability, redox potential). A broader exploration of different property types would enhance the general applicability.

Reviewer #1 (Remarks on code availability):

The code works well and reproduces the results.

Reviewer #2 (Remarks to the Author):

The authors developed a novel molecular descriptor, TDiMS, which demonstrates strengths in describing large molecules. However, several detailed points should be clarified before the manuscript is acceptable for Nature Computational Science.

Major Comment 1: Clarification on SHAP Value Comparison in Figure 3
In Figure 3, the authors compare the proportion of the total absolute SHAP values for TDiMS and Mordred, but the explanation lacks sufficient detail.
First, if the number of features differs between the descriptors, comparing the total absolute SHAP values could be misleading. A fairer comparison might involve averaging the absolute SHAP values across features (sample-wise).
Additionally, the authors state they employed LASSO. However, LASSO lacks group effect handling and introduces randomness when selecting among correlated features. Thus, without using techniques such as stability selection, it is difficult to judge whether their LASSO model appropriately reflects feature importance.
Overall, the authors should provide a more detailed description of the SHAP analysis procedure, including how they accounted for these potential issues.

Major Comment 2: Fairness of Feature Importance Comparison
In Figure 3, the authors compare TDiMS with other descriptors. However, TDiMS appears to extract information directly from the input dataset, while Mordred, MolFormer, and, depending on the setting, MolCLR may not (MolCLR might adapt to the input dataset in the MLP setting, which is too strong.)
To ensure a fair comparison, the authors should clearly specify:
- Which layer and setting were used for feature extraction in each model.
- Whether other descriptors were fine-tuned or adapted to the input dataset.

If TDiMS was adapted but the others were not, additional comparisons are needed, for instance by continual pretraining of MolFormer on the input dataset.
On the other hand, if MolFormer's applicability domain is restricted to low molecular weight compounds and struggles with CPT, the authors should highlight this limitation to further underscore TDiMS's strengths.

Major Comment 3: Clarify Relationship with MolBAR
The recently reported MolBAR method (Digital Discovery, 2024, 3, 2298–2319) also leverages topological information, resembling TDiMS in some aspects.
The authors should discuss the differences between TDiMS and MolBAR, especially regarding topological encoding strategies and adaptation for large molecules, as this would be highly informative for readers. Or if this comment is totally wrong, please let me know it with clear explanations.

Minor Comment 1:
Regarding the sentence:
> "First, the features must reflect essential molecular characteristics for developing an effective property prediction model. This is especially important for large molecules that involve complex intramolecular interactions on nonlocal substructures."
The concern about reflecting essential molecular characteristics is important for both small and large molecules. Instead of emphasizing "importance," the authors should emphasize the greater difficulty for large molecules, due to their more complex intramolecular interactions.

Minor Comment 2:
In line 68:
> "This constraint poses challenges for the effectiveness of descriptors, especially those based on NNs, which typically require larger datasets."

This statement is partially inaccurate. Modern NN-based approaches often utilize self-supervised learning as the authors commented, significantly reducing their dependency on large labeled datasets in extracting features if the adaptation to the input dataset is not necessary. The authors should revise this statement to reflect this nuance.

Minor Comment 3:
The given code link was dead on Apr 29th.

Reviewer #2 (Remarks on code availability):

I checked it on April 29th, but the link was dead.

Version 1:

Decision Letter:

Our ref: NATCOMPUTSCI-25-0634A

3rd October 2025

Dear Dr. Hamada,

Thank you for submitting your revised manuscript "Revisiting Molecular Descriptors with TDiMS for Interpretable Intramolecular Interactions Based on Substructure Pairs" (NATCOMPUTSCI-25-0634A). It has now been seen by the original referees and their comments are below. The reviewers find that the paper has improved in revision, and therefore we'll be happy in principle to publish it in Nature Computational Science, pending minor revisions to satisfy the referees' final requests and to comply with our editorial and formatting guidelines.

We are now performing detailed checks on your paper and will send you a checklist detailing our editorial and formatting requirements in about a week. Please do not upload the final materials and make any revisions until you receive this additional information from us.

TRANSPARENT PEER REVIEW
Nature Computational Science offers a transparent peer review option for original research manuscripts. We encourage increased transparency in peer review by publishing the reviewer comments, author rebuttal letters and editorial decision letters if the authors agree. Such peer review material is made available as a supplementary peer review file. **Please remember to choose, using the manuscript system, whether or not you want to participate in transparent peer review.**
Please note: we allow redactions to authors' rebuttal and reviewer comments in the interest of confidentiality. If you are concerned about the release of confidential data, please let us know specifically what information you would like to have removed. Please note that we cannot incorporate redactions for any other reasons. Reviewer names will be published in the peer review files if the reviewer signed the comments to authors, or if reviewers explicitly agree to release their name. For more information, please refer to our FAQ page.

Thank you again for your interest in Nature Computational Science. Please do not hesitate to contact me if you have any questions.

Sincerely,

Kaitlin McCardle, PhD
Senior Editor
Nature Computational Science

ORCID
IMPORTANT: Non-corresponding authors do not have to link their ORCIDs but are encouraged to do so. Please note that it will not be possible to add/modify ORCIDs at proof. Thus, please let your co-authors know that if they wish to have their ORCID added to the paper they must follow the procedure described in the following link prior to acceptance:
https://www.springernature.com/gp/researchers/orcid/orcid-for-nature-research

Author names using non-Roman characters
Nature Portfolio journals can support presentation of author names using non-Roman characters in the HTML version of the article. If you wish to, please include author names in parentheses after the Roman-character spelling; see example online here. Currently supported scripts are: Arabic, Chinese, Cyrillic, Devanagari, Greek, Hebrew, Hangul, Japanese and Persian. You will be asked to verify the rendering is correct at proof stage.

Reviewer #1 (Remarks to the Author):

Reviewer raised fundamental questions about the scientific validity and interpretability of TDiMS, especially regarding its relation to optical properties, substructure definition, and applicability beyond chromophores. These were deep, conceptual concerns that required substantive revisions.
The authors have fully and convincingly addressed Reviewer's concerns. The rebuttal demonstrates substantial additional work (new calculations, analyses, and clarifications) that strengthens the manuscript both scientifically and in presentation. Reviewer's major concerns are now satisfactorily resolved. The manuscript is ready for acceptance.

Reviewer #1 (Remarks on code availability):

https://ibm.box.com/s/dezvoy3tkj2uwvjgnv1veb4wmqmmxz6g

The above site is not accessible.

Reviewer #2 (Remarks to the Author):

The authors appropriately responded to the comments.
I apologize for my earlier misunderstanding regarding whether the proposed model is adaptive.

Nice work.

Version 2:

Decision Letter:

Our ref: NATCOMPUTSCI-25-0634B

15th May 2026

Dear Dr. Hamada,

Thank you for submitting your revised manuscript "Revisiting Molecular Descriptors with TDiMS for Interpretable Intramolecular Interactions Based on Substructure Pairs" (NATCOMPUTSCI-25-0634B). It has now been seen by one of the original referees and their comments are below.

We ask that you address the following points:

1) Please remove the placeholder for Figure 1

2) Please note that our art editors are checking your chemdraw structures. We will be in touch with redrawn versions, if needed.

3) Please remove "new" from the last paragraph of page 8.

4) Please rename supplementary sections to "Supplementary Section #" rather than A, B, etc

5) Please rename the source data for Supplementary Figures 1-8, so that it's clear that they are source data.

6) Please provide source data for Supplementary Figures 7 and 8 as csv or xlsx (not .log files)

TRANSPARENT PEER REVIEW
Nature Computational Science offers a transparent peer review option for original research manuscripts. We encourage increased transparency in peer review by publishing the reviewer comments, author rebuttal letters and editorial decision letters if the authors agree. Such peer review material is made available as a supplementary peer review file. **Please remember to choose, using the manuscript system, whether or not you want to participate in transparent peer review.**
Please note: we allow redactions to authors' rebuttal and reviewer comments in the interest of confidentiality. If you are concerned about the release of confidential data, please let us know specifically what information you would like to have removed. Please note that we cannot incorporate redactions for any other reasons. Reviewer names will be published in the peer review files if the reviewer signed the comments to authors, or if reviewers explicitly agree to release their name. For more information, please refer to our FAQ page.

Thank you again for your interest in Nature Computational Science. Please do not hesitate to contact me if you have any questions.

Sincerely,

Kaitlin McCardle, PhD
Senior Editor
Nature Computational Science

ORCID
IMPORTANT: Non-corresponding authors do not have to link their ORCIDs but are encouraged to do so. Please note that it will not be possible to add/modify ORCIDs at proof. Thus, please let your co-authors know that if they wish to have their ORCID added to the paper they must follow the procedure described in the following link prior to acceptance:
https://www.springernature.com/gp/researchers/orcid/orcid-for-nature-research

Author names using non-Roman characters
Nature Portfolio journals can support presentation of author names using non-Roman characters in the HTML version of the article. If you wish to, please include author names in parentheses after the Roman-character spelling; see example online here. Currently supported scripts are: Arabic, Chinese, Cyrillic, Devanagari, Greek, Hebrew, Hangul, Japanese and Persian. You will be asked to verify the rendering is correct at proof stage.

Reviewer #1 (Remarks to the Author):

No further review is necessary.

Reviewer #1 (Remarks on code availability):

No further review is necessary.

Version 3:

Decision Letter:

Dear Dr Hamada,

We are pleased to inform you that your Brief Communication "Revisiting Molecular Descriptors with TDiMS for Interpretable Intramolecular Interactions Based on Substructure Pairs" has now been accepted for publication in Nature Computational Science.

Once your manuscript is typeset, you will receive an email with a link to choose the appropriate publishing options for your paper and our Author Services team will be in touch regarding any additional information that may be required.

Authors may need to take specific actions to achieve compliance with funder and institutional open access mandates. If your research is supported by a funder that requires immediate open access (e.g. according to Plan S principles or the NIH public access policy) then you should select the gold OA route, and we will direct you to the compliant route where possible. Because authors warrant under our subscription licensing terms that they haven't committed to licensing any version of their article under a licence inconsistent with the terms of our agreement – including the applicable embargo period – publication under the subscription model isn't suitable for authors whose funders require no embargo.

If you have any questions about our publishing options, costs, Open Access requirements, or our legal forms, please contact ASJournals@springernature.com

Acceptance of your manuscript is conditional on all authors' agreement with our publication policies (see https://www.nature.com/natcomputsci/for-authors). In particular your manuscript must not be published elsewhere and there must be no announcement of the work to any media outlet until the publication date (the day on which it is uploaded onto our web site).

Before your manuscript is typeset, we will edit the text to ensure it is intelligible to our wide readership and conforms to house style. We look particularly carefully at the titles of all papers to ensure that they are relatively brief and understandable.

Once your manuscript is typeset, you will receive a link to your electronic proof via email with a request to make any corrections within 48 hours. If, when you receive your proof, you cannot meet this deadline, please inform us at rjsproduction@springernature.com immediately.

If you have queries at any point during the production process then please contact the production team at rjsproduction@springernature.com.

You may wish to make your media relations office aware of your accepted publication, in case they consider it appropriate to organize some internal or external publicity. Once your paper has been scheduled you will receive an email confirming the publication details. This is normally 3-4 working days in advance of publication. If you need additional notice of the date and time of publication, please let the production team know when you receive the proof of your article to ensure there is sufficient time to coordinate. Further information on our embargo policies can be found here: https://www.nature.com/authors/policies/embargo.html

An online order form for reprints of your paper is available at https://www.nature.com/reprints/author-reprints.html. All co-authors, authors' institutions and authors' funding agencies can order reprints using the form appropriate to their geographical region.

We welcome the submission of potential cover material (including a short caption of around 40 words) related to your manuscript; suggestions should be sent to Nature Computational Science as electronic files (the image should be 300 dpi at 210 x 297 mm in either TIFF or JPEG format). We also welcome suggestions for the Hero Image, which appears at the top of our home page; these should be 72 dpi at 1400 x 400 pixels in JPEG format. Please note that such pictures should be selected more for their aesthetic appeal than for their scientific content, and that colour images work better than black and white or grayscale images. Please do not try to design a cover with the Nature Computational Science logo etc., and please do not submit composites of images related to your work. I am sure you will understand that we cannot make any promise as to whether any of your suggestions might be selected for the cover of the journal.

You can now use a single sign-on for all your accounts, view the status of all your manuscript submissions and reviews,

access usage statistics for your published articles and download a record of your refereeing activity for the Nature journals.

To assist our authors in disseminating their research to the broader community, our SharedIt initiative provides you with a unique shareable link that will allow anyone (with or without a subscription) to read the published article. Recipients of the link with a subscription will also be able to download and print the PDF.

As soon as your article is published, you will receive an automated email with your shareable link.

We look forward to publishing your paper.

Best regards,

Kaitlin McCardle, PhD
Senior Editor
Nature Computational Science

P.S. Click on the following link if you would like to recommend Nature Computational Science to your librarian: https://www.springernature.com/gp/librarians/recommend-to-your-library

** Visit the Springer Nature Editorial and Publishing website at www.springernature.com/editorial-and-publishing-jobs for more information about our career opportunities. If you have any questions please click here.**

Open Access This Peer Review File is licensed under a Creative Commons Attribution 4.0 International License, which permits use, sharing, adaptation, distribution and reproduction in any medium or format, as long as you give appropriate credit to the original author(s) and the source, provide a link to the Creative Commons license, and indicate if changes were made.
In cases where reviewers are anonymous, credit should be given to 'Anonymous Referee' and the source.
The images or other third party material in this Peer Review File are included in the article’s Creative Commons license, unless indicated otherwise in a credit line to the material. If material is not included in the article’s Creative Commons license and your intended use is not permitted by statutory regulation or exceeds the permitted use, you will need to obtain permission directly from the copyright holder.
To view a copy of this license, visit https://creativecommons.org/licenses/by/4.0/

Response to Reviewers

We thank you for your constructive feedback on our manuscript entitled *"Revisiting Molecular Descriptors with TDiMS for Interpretable Intramolecular Interactions Based on Substructure Pairs"* (NATCOMPUTSCI-25-0634).

Our revised manuscripts highlight changes in blue and red and address all points raised in the reviews. Below, we provide point-by-point responses to the reviewers, where each comment is labelled as "Comment X-Y" directly followed by our response.

Responses to Reviewer #1

Comment 1-1: Absorption maximum is more related to how the electron is delocalized in the molecule and how the electron density is changed upon light absorption. It is unclear how TDiMS can be used to understand absorption maximum.

Response:

We revised the description in the Results section for clarification and included an additional analysis in the Supplementary Material which is also related to comments 1-5 and 1-6. Please see the final paragraph of the subsection titled "TDiMS features and physicochemical phenomena" and Supplementary Section C.5.

As pointed out in the comment, absorption maximum is related to the change in the electron density when light is absorbed. How the electron density changes for a point of a compound depends on the chemical structure. Extended Data Figure 4 (also available as Supplementary Figure S6) demonstrates that TDiMS successfully extracts the substructure pairs where electrons have been increased or decreased and the travel distance of the electrons between these pairs leads to improved accuracy for the absorption maximum.

Comment 1-2: In the manuscript, substructures in a given molecule seem to play a critical role in predicting molecular properties. It is unclear how the substructures can be defined in given molecules.

Response:

First, we added the following description in the Methods section (see the beginning of the TDiMS details subsection):

"We use a standard definition for a molecular structure represented as a graph that consists of labelled nodes and edges without three-dimensional coordinates, where a node and an edge correspond to an atom and a bond, respectively. A graph G_{sub} is defined as a substructure to a molecular structure G if G_{sub} is a subgraph of G. G_{sub} is merely called a substructure if G_{sub} is intended to be used for identifying a substructure in a molecule."

Given a molecule, we note that TDiMS *uniquely* determines substructure pairs to that molecule. This is because TDiMS does *not fragment a molecule* into a set of substructure pairs but *enumerates all locations of the substructure pairs* by so-called deterministic algorithms, namely the Morgan fingerprint algorithm, subgraph matching with customised fragments (i.e., CEP), and checking locations of heteroatoms. While there are multiple ways to fragment a molecule, enumerating all locations *always* provides one unique result. The other enumerative descriptors also hold such uniqueness.

To clarify the uniqueness of the results and the set of substructure pairs, we added the following sentence to the second last paragraph of the TDiMS details subsection:

"The final set of enumerated substructure pairs for the dataset depends on the set of molecules in the dataset. However, TDiMS uniquely determines the substructure pairs of each molecule."

Comment 1-3: For a given molecule, are the substructures the same for prediction of absorption maximum, and HOMO and LUMO energies?

Response:

Since QM9 and Chromophore λ_{max} contain different molecules, the substructures extracted from these datasets are different. For clarity, as mentioned in our response to comment 1-2, we included a sentence that explicitly describes this point in the TDiMS details subsection.

Comment 1-4: Depending on the molecular property prediction, it is unclear whether the size (range) of substructures can be varied or not. For example, for prediction of aqueous solubility, optical properties, PLQY, and so on, are the substructures the same or different?

Response:

As mentioned in our responses to comments 1-2 and 1-3, the substructure sets are different, depending on the dataset and the target property to predict. Additionally, we emphasise two important steps of training an estimator with TDiMS described in the Methods section, which is related to other comments on the substructure size.

- **Selection of the best configuration** (see the final paragraph in the TDiMS details subsection):
  "As mentioned above, there are several possible combinations of features for TDiMS. For all combinations of configurations based on (1) the inclusion or exclusion of CEP fragments, (2) the use of circular substructures from the Morgan fingerprints with radii of $r = 1$ *or* 2, *(3) the selection of* $f_{dis} = x^{-2}$, x^{-1} *or* x, *and (4)* f_{dup} = *sum, max or min, TDiMS attempts to perform some initial training, and the most promising combination is passed to the estimator. Heteroatom features are always included."*
- **Feature selection** (the first paragraph in the subsection titled "Details on the prediction models"):
  "Feature selection and hyperparameter optimization (HPO) are known to yield significant improvements in practice. In feature selection, an effective subset of features passed to the estimator is selected."

These two steps enable *an appropriate substructure size to be automatically selected* (e.g., the best radius of Morgan fingerprint) as well as *select substructures that contribute to performance improvement*, thus successfully building an optimised prediction model.

By using these two steps, Extended Data Table 1 (also available as Supplementary Table S1) and Supplementary Section A.2 show that the substructure sets are adapted to fit well into the molecules in the dataset and their target property. For example, with the above first step to select the best configuration, Extended Data Table 4 (also available as Supplementary Table S6) and Supplementary Section C.2 indicate that the substructure set of TDiMS includes only small substructures (i.e.,

Morgan fingerprint of radius 1) for the dataset with small molecules. Please refer to the following sentences in Supplementary Section C.2:

"For the Chromophore datasets, the radius of the MFP was set to 2, indicating that larger substructures need to be considered. In contrast, the radius for QM9 was set to 1, which was attributed to its small substructures."

For further clarification, we also included additional discussions in Supplementary Section C.3 and the Results section (see the final paragraph of the subsection titled "Insights and validity of TDiMS features").

Comment 1-5: The optical properties (absorption max) of organic compounds are determined by the frontier orbitals (HOMO and LUMO), which can be localized in certain substructures or delocalized in entire molecules depending on the molecular structures. It is unclear how TDiMS is related to the optical properties.

Response:

We performed the DFT and TD-DFT calculations for the four molecules related to the absorption maxima and substructure pairs shown in the left of Figure 5a. Even if the optimal transition mode depends on the molecular structure, our results on comparing DFT calculations with the substructure pairs identified by TDiMS demonstrate that TDiMS can flexibly capture various substructures involved in different optical transition modes. Please see Extended Data Figure 4 (also available as Supplementary Figure S6) and Supplementary Section C.5 for detailed discussions relating the substructure pairs of TDiMS to the transitions on the molecular orbitals.

Comment 1-6: Absorption by large molecules (donor-acceptor types) induces strong intramolecular charge transfer (ICT) within the molecules. It is unclear how TDiMS can be used to explain the ICT.

Response:

As a result of the DFT calculations, we found the first molecule at the top of the left panel in Figure 5a involved a strong ICT that is typical for donor-acceptor types. Please see Extended Data Figure 4 (also available as Supplementary Figure S6) and Supplementary Section C.5 for details.

Comment 1-7: To predict HOMO and LUMO energies of small molecules (QM9 dataset), how the substructures are defined? How the predicted HOMO and LUMO energies are varied depending on the size (range) of substructures?

Response:

Please see our responses to comments 1-2, 1-3, and 1-4.

Comment 1-8: Can TDiMS be applicable to predict other molecular properties excluding optical properties and HOMO and LUMO energies, such as aqueous solubility, logP, glass temperature of polymers, and so on?

Response:

From a viewpoint of material and chemical sciences, we believe TDiMS has an advantage in prediction tasks where the distance of substructure pairs is an essential factor for calculating molecular properties. To further support this hypothesis, we performed one more property prediction task on dipole moment for which TDiMS successfully outperformed the other descriptors. We newly included the results in

Supplementary Section B.5 and in the Results section (the subsection titled "Analysis of the heavy atom count and effectiveness of TDiMS for dipole moment prediction") in the main manuscript. Please also refer to our response to comment 1-9 as a topic related to dipole moment.

TDiMS can be algorithmically applied to predict other molecular properties in general. However, our main focus is Chromophore and their optical properties. While these other properties mentioned in the comments are important for both chemical and material sciences, follow-up research needs to be performed. We, therefore, updated the manuscript and mentioned these tasks as future work in the Discussion section.

Comment 1-9: The authors should provide a more detailed discussion or quantitative thresholds for when TDiMS is no longer advantageous (e.g., heavy atom counts, molecular weight limits).

Response:

We introduced the new subsection titled "Analysis of the heavy atom count and effectiveness of TDiMS for dipole moment prediction" to reflect this comment in our revision. In this subsection, we created datasets each of which contains 1000 molecules with a specified range of the heavy atom count, extracted from the PubChemQC dataset, and empirically assessed the quantitative threshold on the heavy atom count of TDiMS for dipole moment prediction. Our results show that the advantage of TDiMS appeared when using the datasets where molecules had at least 25 heavy atoms.

We also note that performing the analysis on the quantitative threshold requires a reasonable number of molecules of both small and large heavy atom counts, which prevents us from performing similar evaluations for each of QM9 (i.e. only molecules with up to nine heavy atoms can be used) and Chromophore (i.e., only large molecules can be used).

Comment 1-10: TDiMS relies heavily on the choice of substructure (CEP fragments, Morgan fingerprints). The method's sensitivity to substructure selection is not fully explored. Would alternative fragmentations (e.g., random, functional groups) lead to similar or worse performance? Robustness to this choice should be discussed.

Response:

Please see our response to comment 1-4, especially regarding related descriptions in Supplementary Sections C.2 and C.3 including Tables S6–S8 (also available as Extended Data Tables 4-6) as well as the final paragraph of the subsection titled "Insights and validity of TDiMS features" in the Results section. In our revision especially to the Supplementary Material, we discussed and emphasised the fact that our approach to automatically select the most promising configuration successfully avoided choosing low-performing combinations of the substructures, thus resulting in TDiMS robustly selecting effective substructures.

Alternative fragmentations might indicate either (1) fragmenting a molecule into a set of substructures or (2) creating a set of customised fragments rather than CEP. Our responses to these two approaches are summarised as follows:

- For the former approach, TDiMS does not fragment the molecule into a set of substructures but enumerates all locations of the substructure pairs (please

see our response to comment 1-2). Therefore, this approach is not related to TDiMS and cannot be applied.

- For the latter approach, because no appropriate fragment database is available for dipole moment prediction, we applied the MacFrag method directly to the PubChemQC datasets and created a custom substructure list that was more suitable for larger molecules (see Supplementary Figure S4 for the list of substructures). Supplementary Section C.3 demonstrates that including this custom substructure list led to additional improvements over TDiMS with Morgan fingerprint $r = 2$, when molecules had at least 17 heavy atoms (also see Supplementary Table S8, which is also available as Extended Data Table 6). Furthermore, Supplementary Table S9 with the custom substructure set, CEP, and BRICS clearly shows that using an appropriate fragment dataset was important.

On the other hand, creating a fragment dataset has been an important research topic and remains an open issue in general. Even for simple approaches such as random and functional groups, there are too many choices in algorithm designs, such as how many fragments to include; what are the maximum number of atoms, double bonds, and triple bonds to include in each fragment; and what kinds of functional groups to include. A significant amount of follow-up research will be needed, which will provide new opportunities for the research communities. Therefore, we mention that establishing an approach to create the custom substructure set is important future work in the Discussion section.

Comment 1-11: The authors claimed that TDiMS captures photophysical phenomena (e.g., donor–acceptor distance, conjugation length). However, it is unclear whether TDiMS generalizes equally well to other types of properties (e.g., mechanical strength, thermal stability, redox potential).

Response:

As mentioned in our response to comment 1-8, while TDiMS itself is a generic algorithm which can be used for predicting other types of properties, the performance evaluation for these types is beyond the scope of our manuscript. In the revision, we clearly state that this task as an important direction of future work (see the revised Discussion section).

On the other hand, we claim that TDiMS shows an advantage for prediction tasks where the distances of substructure pairs are an essential factor for calculating molecular properties, which is also evidenced by an additional performance evaluation on dipole moment.

Responses to Reviewer #2

Comment 2-1: In Figure 3, the authors compare the proportion of the total absolute SHAP values for TDiMS and Mordred, but the explanation lacks sufficient detail. First, if the number of features differs between the descriptors, comparing the total absolute SHAP values could be misleading. A fairer comparison might involve averaging the absolute SHAP values across features (sample-wise).

Response:

The number of features depends on the descriptor. We agree that the total absolute SHAP values for the member features could unrightfully favour the descriptors with

many feature dimensions. In particular, if multiple features have SHAP values of alternating signs in a descriptor, the sum of these absolute SHAP values might inflate the importance score of the parent descriptor even if these values can collectively cancel out each other and do not contribute much to the model's prediction.

In the revised version, we re-performed the analysis for Figures 3b and 3c with SHAP for groups named *group SHAP* (gSHAP). For gSHAP, the descriptor importance score is defined as the magnitude of the sum of *raw* SHAP values for the member features.

Our gSHAP i) resolves the aforementioned problem of importance overestimation and more importantly ii) generalises the original SHAP within a standard explainable-AI framework known as *additive explanation*. As a result, the revised score ensures interpretability as the net additive effect of the descriptor's presence in the model's prediction. On the other hand, this is not the case for our previous scoring method or the one obtained by averaging the absolute SHAP values across features.

The updated versions of Figures 3b and 3c with gSHAP show the same tendency that TDiMS' contributions were significant for Chromophore while Mordred contributed more for QM9. Please see the revised description in the subsection titled "Effectiveness of TDiMS when combined with other descriptors" for details.

We also newly report the (non-grouped) SHAP-value distribution of each descriptor for more detailed evaluation on feature importance in Supplementary Section B.4. As observed in Supplementary Figure S2, TDiMS exhibits a more heavy-tailed SHAP distribution than Mordred/MolCLR does, especially with the Chromophore datasets. In other words, it is not the case where TDiMS only has an extremely large number of mediocre features. TDiMS does provide more important features than Mordred when molecules in the dataset contain substructure pairs suitable for TDiMS.

Comment 2-2: The authors state they employed LASSO. However, LASSO lacks group effect handling and introduces randomness when selecting among correlated features. Thus, without using techniques such as stability selection, it is difficult to judge whether their LASSO model appropriately reflects feature importance.

Response:

We agree that the stability of the learned parameters and the reliability of the resultant analysis need to be ensured. Therefore, we addressed these points with bootstrapped sampling in ten trials and revised the subsection titled "Effectiveness of TDiMS when combined with other descriptors", which includes the new version of Figure 3 (please see the caption which describes how bootstrap sampling was performed too). The same phenomenon described in the submitted version was observed in the revised results. Please also see our response to comment 2-1.

Comment 2-3: Overall, the authors should provide a more detailed description of the SHAP analysis procedure, including how they accounted for these potential issues.

Response:

In summary, we re-performed the evaluation with the raw SHAP values and bootstrap sampling and revised the main and supplementary manuscripts accordingly. Please also see our responses to comments 2-1 and 2-2.

Comment 2-4: In Figure 3, the authors compare TDiMS with other descriptors. However, TDiMS appears to extract information directly from the input dataset, while Mordred, MolFormer, and, depending on the setting, MolCLR may not (MolCLR might adapt to the input dataset in the MLP setting, which is too strong.)

Response:

We emphasise that the substructure extraction step of TDiMS is *neither data adaptation nor pre-training of a machine learning model*, but is merely an algorithmic trick for computational efficiency in time and space. From the machine learning perspective, the extraction step does *not* lead to any inappropriate favour to TDiMS over the other descriptors.

To illustrate the above, we show the fact that the output of TDiMS does not change with or without the extraction step.

Consider a theoretical implementation of TDiMS without the substructure extraction step. The feature set of this theoretical implementation has a one-to-one correspondence with a large (but finite) predefined set of substructure pairs, e.g., all the pairs of substructures with the radii no more than 2. However, for a given training dataset, many features in this theoretical set never activate throughout the training phase because they are not included in any molecules in the dataset.

Our extraction step is designed to truncate such features. This truncation does not influence the prediction accuracy or the SHAP-based scoring, since the truncated feature dimensions always have value zero and such features never contribute to the outputs of linear models. Therefore, both theoretical and actual implementations always return identical scores, irrespective of the presence or absence of the substructure extraction step.

In summary, TDiMS is no more adaptive than Mordred and the other methods in the performance evaluation since the underlying feature set is fixed before training.

Comment 2-5: To ensure a fair comparison, the authors should clearly specify: (i) Which layer and setting were used for feature extraction in each model. (ii) Whether other descriptors were fine-tuned or adapted to the input dataset.

Response:

Regarding (i), we revised the Methods section to clarify the descriptions on the embedding-only and fine-tuned approach for the NN-based descriptors (see the MoLFormer and MolCLR subsections for details). Additionally, we corrected the description on fine-tuning MolCLR which updates all model parameters and newly included the fine-tuned version of MoLFormer which also updates all model parameters. We note that this is the state-of-the-art fine-tuning approach for CLMs. Whether other efficient fine-tuning methods commonly used by LLMs (e.g., LoRA) are applicable to CLMs like MoLFormer remains an open question.

Regarding (ii), we revised the subsection titled "Setups for physical property prediction tasks" and clearly described that we used that we considered both embedding-only and fine-tuned settings for MolCLR and MoLFormer to evaluate predictive performance and that the fine-tuned models are comparable to the others only in terms of the predictive performance. The caption for Figure 3 also clarifies that only the embedding-only setting (i.e., frozen models) was used to analyse feature contributions, while the captions for Table 1 and Extended Data Table 2 clarify that the better score between embedding-only and fine-tuned settings in each case is included.

Additionally, we revised the results related to the usage of fine-tuned MoLFormer, e.g., Table 1, Extended Data Figures 1, Extended Data Table 2 (also available as Supplementary Table S2), etc. accordingly.

We note that the results on MolCLR remain the same because they were already obtained by the fine-tuned version that updates all model parameters.

Please also see our response to comment 2-6 for the detailed reasons that we employed the frozen models for Figure 3.

Comment 2-6: If TDiMS was adapted but the others were not, additional comparisons are needed, for instance by continual pretraining of MolFormer on the input dataset. On the other hand, if MolFormer's applicability domain is restricted to low molecular weight compounds and struggles with CPT, the authors should highlight this limitation to further underscore TDiMS's strengths.

Response:

We tried our best to make fair comparisons among all descriptors, while considering other factors such as the availabilities of the computational resources and limitations to the state-of-the-art approaches.

As mentioned in our response to comment 2-4, from the machine learning perspective, the substructure extraction step of TDiMS is neither data adaptation nor pre-training.

For Figure 3 including the SHAP-based analysis, we employed the frozen models for the NN-based descriptors for the following reasons:

- It is nontrivial to combine the descriptor-based linear models and the end-to-end neural models induced by fine-tuning/CPT of NNs in a way that allows us to explain each submodel's contribution to the prediction of the combined model. For instance, SHAP is a black-box explanation method that only explains the prediction of a model in terms of its input. However, unfortunately, the input types for the descriptor-based and end-to-end models are completely different as the former takes an intermediate feature vector and the latter takes raw structural data as their inputs. Thus, conventional black-box explanation methods cannot be straightforwardly applied to compare the importance of models with such different input types.
- Even if the aforementioned problem is resolved, fine-tuning requires a non-negligible amount of computational time and monetary cost because of a large number of parameters updated in training. This is especially the case for MoLFormer. For example, filling out numbers for Table 1 on MoLFormer roughly required a total of 300 GPU hours with our accessible computational resource that has NVIDIA H100 GPUs.

Comment 2-7: The recently reported MolBAR method (Digital Discovery, 2024, 3, 2298–2319) also leverages topological information, resembling TDiMS in some aspects. The authors should discuss the differences between TDiMS and MolBAR, especially regarding topological encoding strategies and adaptation for large molecules.

Response:

We revised the Discussion section and included one paragraph which concludes that combining TDiMS and MolBar is important future work.

MolBar and TDiMS take different approaches for extracting global molecular structures. One crucial difference is that MolBar is a knowledge-driven approach which preprocesses the molecular graphs into the eigenspectra of modified adjacency matrices and compresses the global topological information in relatively few features.

Although MolBar is a unique molecular identifier that supports full streochemistry as its advantage and can also be regarded as a molecular descriptor, applying MolBar to property prediction remains an open issue, and its compression technique might potentially lead to the loss of representing important features in predicting properties. On the other hand, TDiMS does not compress the information but enumerates all the possible substructure pairs. Then, TDiMS lets the downstream ML model select the useful ones, which has led to it more accurately predicting properties where topological distances between substructures are a primary factor.

Comment 2-8: The concern about reflecting essential molecular characteristics is important for both small and large molecules. Instead of emphasizing "importance," the authors should emphasize the greater difficulty for large molecules, due to their more complex intramolecular interactions.

Response:

We updated the sentence as follows (see the Introduction section):

"This is especially difficult for large molecules because of their complex intramolecular interactions on nonlocal substructures."

Comment 2-9: The statement on line 68 that "NNs typically require large datasets" is partially inaccurate. Modern NN-based approaches often utilize self-supervised learning, which reduces this dependency.

Response:

We removed the above phrase which sounded unfavourable to NNs and merely mentioned this constraint is a challenge for the descriptors in general as follows (see the Introduction section):

"An additional challenge for creating effective descriptors is that the target property datasets used to train ML estimators are limited to specific molecules."

Comment 2-10: The given code link was dead on Apr 29th.

Response:

We found the previous link was active only for a certain period. The updated and verified code repository can be accessed at the following URL:

`https://ibm.box.com/s/pmyis9mpvj04rretv227eitgagk6c48u`

We hope this resolves the issue.

We thank you in advance for your time and effort in reviewing our manuscripts again. We hope that our resubmission is now acceptable and look forward to hearing from you.

Sincerely,
Lisa Hamada (on behalf of all authors)

Response to the Reviewers

Manuscript title: *Revisiting Molecular Descriptors with TDiMS for Interpretable Intramolecular Interactions Based on Substructure Pairs*

We sincerely thank the reviewers for their positive assessment of our revised manuscript.

After the review process, we identified a potential data leakage issue in the evaluation pipeline during cross-validation in the hyperparameter optimization process of the prediction models. We therefore reconducted all affected experiments using a corrected pipeline. As described in the Cover Letter, the revised version completely resolves the issue, and the updated results do not alter the central conclusions regarding the capabilities and scientific value of TDiMS.

Reviewer #1

Reviewer comment:
Reviewer raised fundamental questions about the scientific validity and interpretability of TDiMS, especially regarding its relation to optical properties, substructure definition, and applicability beyond chromophores. These were deep, conceptual concerns that required substantive revisions. The authors have fully and convincingly addressed Reviewer's concerns. The rebuttal demonstrates substantial additional work (new calculations, analyses, and clarifications) that strengthens the manuscript both scientifically and in presentation. Reviewer's major concerns are now satisfactorily resolved. The manuscript is ready for acceptance.

Response:
We sincerely thank the reviewer for the constructive comments provided in the earlier round of review. We are grateful for the reviewer's positive assessment of our revisions and for recognizing the additional analyses, calculations, and clarifications included in the revised manuscript.

We would also like to note that the TDiMS source code has now been made publicly available at: `https://github.com/IBM/materials/tree/main/models/tdims`.

Reviewer #2

Reviewer comment:
The authors appropriately responded to the comments. I apologize for my earlier misunderstanding regarding whether the proposed model is adaptive. Nice work.

Response:
We sincerely thank the reviewer for the positive feedback and the initial comments which greatly helped improve the manuscript. We also appreciate the reviewer's acknowledgment regarding the clarification of the adaptive nature of the proposed method. We are grateful for the reviewer's supportive assessment.
